# Development of a highly efficient prime editor 2 system in plants

**DOI:** 10.1186/s13059-022-02730-x

**Published:** 2022-07-25

**Authors:** Juan Li, Like Chen, Jing Liang, Rongfang Xu, Yingli Jiang, Yizhen Li, Jian Ding, Min Li, Ruiying Qin, Pengcheng Wei

**Affiliations:** 1 Key Laboratory of Rice Genetic Breeding of Anhui Province, Rice Research Institute, Anhui Academy of Agricultural Sciences, Hefei, 230031 P.R. China; 2 College of Agronomy, Anhui Agricultural University, Hefei, 230036 P.R. China

**Keywords:** Prime editing, Efficiency, Engineering, Rice, Precise editing

## Abstract

Low efficiency has seriously restricted the application of prime editing (PE) systems in plants. In this study, we develop an enhanced plant prime editor 2 system, enpPE2, by stacking various optimization strategies, including updating the PE architecture to PEmax and expressing engineered pegRNA with a structured motif under the control of a composite promoter. In T_0_ rice plants, enpPE2 exhibits editing frequencies of 64.58% to 77.08%, which are much higher than the frequencies with unmodified pPE2. Our results indicate that the enpPE2 system provides a robust and powerful tool for the precise modification of plant genomes.

**Supplementary Information:**

The online version contains supplementary material available at 10.1186/s13059-022-02730-x.

## Background

Prime editing represents a major breakthrough in CRISPR-mediated genetic engineering technology for introducing precise changes in plant genomes. In this system, a chimeric prime editing guide RNA (pegRNA) directs a prime editor (PE) fusion protein composed of a Cas9 nickase and an evolved Moloney murine leukemia virus reverse transcriptase (M-MLV RT) to enable the programmable installation of base substitutions and small indels [1]. Although prime editing has been successfully demonstrated to introduce flexible mutations in several targets in both monocots and dicots, it has been practically applied for crop trait improvement quite rarely due to its limited efficiency [2]. To overcome this limitation, considerable efforts have been devoted to optimize the activity of plant prime editing systems [2]. For example, cells harboring active or highly expressed PEs were enriched to increase the screening efficiency of edited plants [3, 4]; increasing pegRNA levels by doubling the expression cassette or using a U6 composite promoter was shown to increase the editing rate from 0 to 43.8% in maize [5]; tuning the melting temperature of the prime binding site (PBS) sequence in pegRNA to 30°C and editing the target with paired pegRNAs increased the efficiency from 2.9- to 17.4-fold [6]; and an improved PE3 system combining a PE with N-fusion M-MLV and synonymous mutations in the RT template stimulated editing activity in rice, maize, and human cells [7]. Nevertheless, improvements in plant prime editing systems are still urgently needed since the efficiencies of the engineered PEs are highly variable at different sites and are generally far lower than those of base editors.

## Results and discussion

**Fig. 1:**
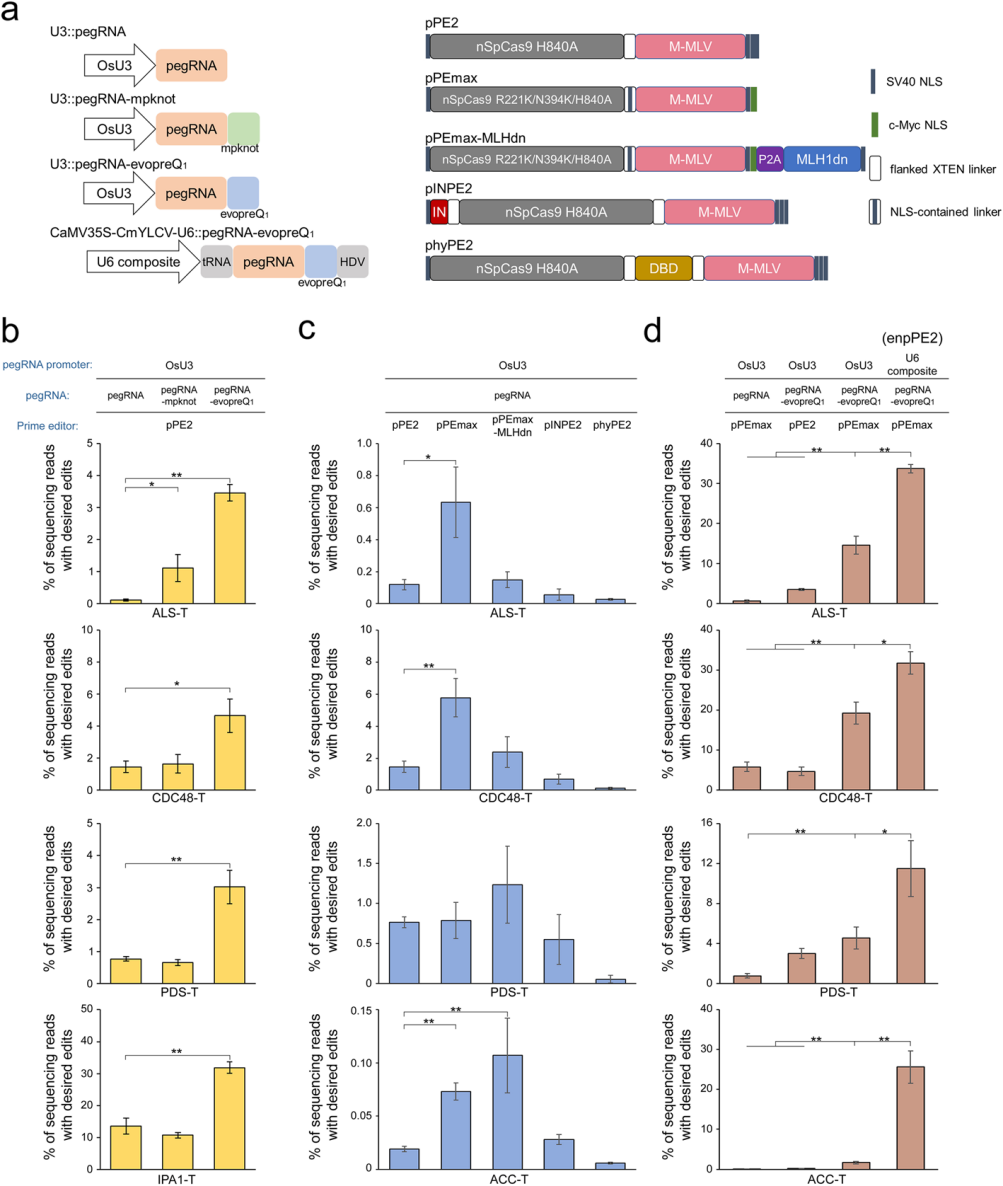
Optimization of plant prime editing systems in rice. **a** Schematic illustration of the engineering of plant prime editing systems. Left, pegRNA expression systems. OsU3, rice U3 promoter; U6 composite, composite promoter of the CaMV 35S enhancer, CmYLCV promoter, and shortened U6-26 promoter; tRNA (Gly-tRNA) and HDV ribozyme were built in the expression cassette driven by the U6 composite promoter to precisely process pegRNAs. Right, engineered prime editors. DBD, hRad51-ssDBD; IN, IGFpm1-NFATC2IPp1 dual peptide; P2A, porcine teschovirus-1 2A self-cleaving peptide; MLH1dn, dominant-negative mutant of the MLH1 protein; NLS, nuclear localization signal. The elements were codon-optimized for rice expression. **b** Editing efficiency of pPE2 in stably transformed callus cells with canonical pegRNAs (pegRNA), pegRNAs engineered by appending the structured RNA motif sequence of mpknot (pegRNA-mpknot) or evopreQ_1_ (pegRNA-evopreQ_1_) at the 3′ end with an 8-nt linker. **c** Editing efficiency of pPE2 variants with canonical pegRNAs in stably transformed callus cells. **d** Editing efficiency of pPEmax with pegRNA-evopreQ_1_ in stably transformed callus cells. The ratios of reads carrying the desired mutations to total clean reads were calculated as the prime editing efficiency. Data are presented as the mean value and standard deviation of three biological replicate samples from independent transformations. Statistical differences were determined by two-tailed *t* tests. *, *p*<0.05; **, *p*<0.01

It has been reported that the degradation of the 3′ terminus of pegRNA impedes prime editing and that incorporating pseudoknots into pegRNA enhances the stability and thus improves the efficiency of the prime editing system by 3~4-fold in several types of human cells [8]. To validate this strategy in plants, a structured RNA motif consisting of either a modified prequeosine1_-1_ riboswitch aptamer (evopreQ_1_) or an M-MLV frameshifting pseudoknot (mpknot) was added at the 3′ end of pegRNA with an 8-nt linker in the previously established plant prime editor 2 (pPE2) system (Fig. 1a) [3]. Four targets within the endogenous *OsCDC48*, *OsIPA1, OsALS*, and *OsPDS* genes were chosen for the introduction of C-to-A, C-to-G, or combined G-to-T and C-to-A conversions or a single T insertion, respectively (Additional file 1: Fig. S1). To evaluate the editing efficiency of engineered pegRNAs (epegRNAs), the pPE2 vectors were transformed into the japonica rice Nipponbare cultivar via Agrobacterium-mediated transformation. The stably transgenic calli were collected and examined by targeted amplicon deep sequencing. We identified 2.35- to 29.22-fold increases in mutation frequencies when using epegRNAs with evopreQ_1_ modification (pegRNA-evopreQ_1_) relative to the canonical pegRNAs across all four sites (Fig. 1b, *p*<0.05). In contrast, significant efficiency improvement was only detected at the ALS-T site when using the mpknot-containing epegRNA (pegRNA-mpknot) instead of canonical pegRNA (Fig. 1b). To further assess epegRNA activity, the vectors targeting ALS-T, CDC48-T and PDS-T were retransformed into rice to generate individual T_0_ plants. Targeted sequencing showed that on average, 13.19%, 17.36%, and 39.58% of the lines were precisely edited in pegRNA, pegRNA-mpknot, and pegRNA-evopreQ_1_ plants, respectively (Table 1). These data confirmed that the epegRNAs could promote pPE2 optimization, although the effects of the RNA motifs may differ among different sites.

**Table 1:** Prime editing by optimized pPE2 systems in transgenic plants

Targets	pPE variants	pegRNA structures	pegRNA promoters^a^	Tested lines^b^	Precisely edited plants^c^			Plants with byproduct^d^
					Ho	He	Total (%)	
ACC-T	pPE2	pegRNA	OsU3	48	0	0	0 (0.00%)	0
	pPE2	pegRNA-evoPreQ_1_	OsU3	48	0	1	1 (2.08%)	0
	pPEmax	pegRNA-evoPreQ_1_	OsU3	48	0	7	7 (14.58%)	0
	pPEmax	pegRNA-evoPreQ_1_	U6 composite	48	0	34	34 (70.83%)	1
PDS-T	pPE2	pegRNA	OsU3	48	0	4	4 (8.33%)	0
	pPE2	pegRNA-mpknot	OsU3	48	0	5	5 (10.42%)	0
	pPE2	pegRNA-evoPreQ_1_	OsU3	48	0	10	10 (20.83%)	0
	pPEmax	pegRNA-evoPreQ_1_	OsU3	48	0	14	14 (29.17%)	0
	pPEmax	pegRNA-evoPreQ_1_	U6 composite	48	10	21	31 (64.58%)	4
ALS-T	pPE2	pegRNA	OsU3	48	0	1	1 (2.08%)	0
	pPE2	pegRNA-mpknot	OsU3	48	0	8	8 (16.67%)	0
	pPE2	pegRNA-evoPreQ_1_	OsU3	48	0	23	23 (47.92%)	0
	pPEmax	pegRNA-evoPreQ_1_	OsU3	48	8	24	32 (66.67%)	0
	pPEmax	pegRNA-evoPreQ_1_	U6 composite	48	16	20	36 (75.00%)	2
CDC48-T	pPE2	pegRNA	OsU3	48	0	14	14 (29.17%)	0
	pPE2	pegRNA-mpknot	OsU3	48	0	12	12 (25.00%)	0
	pPE2	pegRNA-evoPreQ_1_	OsU3	48	0	24	24 (50.00%)	0
	pPEmax	pegRNA-evoPreQ_1_	OsU3	48	8	22	30 (62.50%)	0
	pPEmax	pegRNA-evoPreQ_1_	U6 composite	48	19	18	37 (77.08%)	0

*He*, heterozygous mutants; *Ho*, homozygous mutants

^a^U3, OsU3 promoter; U6 composite, CaMV 35S enhancer-CmYLCV-U6 composite promoter

^b^For each vector, 48 independent lines were randomly selected for genotyping

^c^The number of transgenic plants harboring precise edits

^d^The number of plants carrying undesired mutations in the targeted region

Recent studies suggested that specific DNA mismatch repair (MMR) antagonizes prime editing, while inhibiting MMR by coexpressing a dominant negative MMR protein (MLH1dn) improves the efficiency of PE in various types of mammalian cells [9]. To examine whether prime editing could be improved via a similar strategy in crops, we first updated the PE fusion in the pPE2 system to a pPEmax architecture with an R221K/N394K nSpCas9 (H840A) mutant, a 34 aa NLS-embedded linker, and C-terminal heterogeneous tandem NLSs [9]. Subsequentially, MLH1dn was integrated into the C-terminus of pPEmax to form a pPEmax-MLHdn variant (Fig. 1a). The pPE2max and pPE4max systems were then constructed by replacing the editor protein of the pPE2 system with pPEmax and pPEmax-MLHdn, respectively. Their editing efficiencies were tested at the aforementioned ALS-T, CDC48-T, and PDS-T sites and at a target selected in the *OsACC1* gene for G-to-C mutation in stably transformed rice calli and were quantified by deep sequencing. The data showed that the editing frequency induced by pPE2max was significantly increased by 3.80-, 5.35-, and 3.98-fold compared to that induced by pPE2 at the ACC-T, ALS-T, and CDC48-T sites, respectively (Fig. 1c). Unexpectedly, pPE4max did not significantly increase the editing efficiency compared to either pPE2max at all tested sites or pPE2 at all targets except for ACC-T (*p*<0.05). These data suggested that prime editing was not fortified by MLH1dn overexpression in our assays. Previous reports revealed that additional synonymous mutations in the RT template stimulated prime editing in crop cells, which implied that manipulating MMR could regulate PEs in plants [7]. In this case, our results hinted that human MLH1dn in the pPE4max system might not suppress the MMR activity of plant cells as efficiently as that of mammalian cells. In the future, homogenous MMR inhibitors screened from plants might be more appropriate for engineering pPEs. Moreover, a prime editing-activated dual peptide combined with a phosphomimetic peptide from IGF1 (IGF1pm1) and an N-terminal peptide from NFATC2IP (NFATC2IPp1) were screened from a human DNA repair-related gene-derived peptide library [10]. A plant IGFpm1-NFATC2IPp1-PE2 (pINPE2) tool was constructed by adding the dual peptide to the N-terminus of pPE2 (Fig. 1a). However, we did not observe a marked difference in the editing efficiency of pPE2 and pINPE2 in rice calli (Fig. 1c). A single-stranded DNA-binding domain of human Rad51 (hRad51-ssDBD) that enhanced the efficiency of base editors [11] was also exploited in the modification of plant PEs. A plant hyperactive PE2 (phyPE2) was constructed by fusing the DBD domain between SpCas9 nickase and M-MLV RT (Fig. 1a). Unlike the higher efficiency of DBD-integrated hyPE2 relative to PE2 in human cells [12], we found that phyPE2 conducted less efficient editing than pPE2 at the tested sites in rice (Fig. 1c).

Then, we examined whether the pPE2max system could be further optimized by pegRNA-evopreQ_1_. Deep sequencing analyses showed that the evopreQ_1_ modification on pegRNA consistently enhanced the efficiency of pPE2max (*p*<0.01), from 0.07 to 1.64% at the ACC-T site, from 0.79 to 4.56% at the PDS-T site, from 0.63 to 14.55% at the ALS-T site, and from 5.78 to 19.20% at the CDC48-T site (Fig. 1d). Moreover, the tested targets other than PDS-1 showed 4.13- to 7.52-fold higher editing frequencies when using pegRNA-evopreQ_1_ in the pPE2max vector (pPE2max-evopreQ_1_) rather than pPE2-evopreQ_1_ (*p*<0.01, Fig. 1d). These results showed that pPEmax and pegRNA-evopreQ_1_ engineering exerted synergistic effects on pPE2 optimization. To generate precise editing more efficiently, an enhanced pPE2 (enpPE2) system was developed by replacing the OsU3 promoter with the U6 composite promoter in the pPE2max system (Fig. 1a). This promoter was engineered from a polymerase II CmYLCV promoter by reinforcing a CaMV35S enhancer and a shortened polymerase III U6-26 promoter. Compared with polymerase III promoters, the U6 composite promoter might drive higher expression levels of pegRNAs and thus be more effective in maize genome prime editing [5, 7]. In rice callus cells, enpPE2 showed mutation frequencies of 25.60% at the ACC-T site, 11.50% at the PDS-T site, 33.75% at the ALS-T site, and 31.77% at the CDC48-T site, representing 1.66- to 15.60-fold increases relative to those obtained with the OsU3-driven pPE2max-evopreQ_1_ vectors (Fig. 1d, *p*<0.05). Notably, enpPE2 achieved an average editing frequency of 25.65%, showing an ~43.47-fold efficiency increase (maximum ~1280-fold increase from 0.02% to 25.60% at the ACC-T site) relative to the average 0.59% frequency produced by the unmodified pPE2 system. Consistent with the improved activity of enpPE2 in embryonic calli, 64.58% to 77.08% of the T_0_ lines were precisely edited by enpPE2, which were much higher than the frequencies of 0% to 29.17% achieved with unmodified pPE2 (Table 1). Furthermore, 20.83% to 39.58% of the plants carried the desired homozygous mutation at the PDS-T, ALS-T, and CDC48-T sites, validating the upgraded activity of enpPE2. Editing byproducts, including unintended indels and/or sgRNA scaffold replacements in addition to the desired mutations (Additional file 1: Fig. S2), were detected in a few enpPE2 plants: at the ACC-T site in 1 out of 48 plants (1/48), at the PDS-T site in 4/48, and at the ALS-T site in 2/48 (Table 1).

To further evaluate the performance of enpPE2, 10 individual epegRNAs were designed for genome editing in an elite japonica variety Wuyungeng31. In regenerated plants, the desired C-to-G, C-to-A, T-to-A, T-to-C, G-to-A, G-to-C, A-to-T, and GA-to-CG substitutions, 2-bp (TA) deletion, and 1-bp (G) insertion were generated by the enpPE2 system in 53.13% of lines on average (Additional file 1: Table S1). Homozygous mutations at the targeted positions were identified across 8 out of 10 sites with a maximal frequency of 52.08% at the SPL17-T site, confirming the sufficient editing efficiency of the enpPE2 system in a commercial rice variety. Interestingly, although editing byproducts were not detected or were found at a relatively low frequency below 8.33% at 6 out of 10 sites, 41.67–62.50% of plants exhibited an undesired A-to-G substitution at the position immediately flanked by the RT template at the D2-T, Ehd1-T, NAL1-T, and SERK1-T sites (Additional file 1: Fig. S2 and Table S1), which might have been derived from the first “G” of the sgRNA scaffold next to the RT template in the epegRNA sequence. These results suggest that the byproduct efficiency of enpPE2 may vary greatly among targets. In addition, Pita and Pid3 were simultaneously targeted in Wuyungeng31 by coexpressing two epegRNAs in the enpPE2 system (Additional file 1: Fig. S3). We identified 11 double-mutant lines among 48 independent transgenic lines, demonstrating multiple options of using enpPE2 for prime editing in plants.

## Conclusions

In this study, we tested various strategies for increasing the prime editing efficiency in rice. An enpPE2 system was developed by stacking feasible strategies, such as updating the PE architecture to PEmax and expressing engineered pegRNA with a structured motif under the control of a composite poly II and a small nuclear RNA promoter. Our results indicated that optimized enpPE2 could introduce extraordinarily frequent, precise mutations in transgenic plants. Very recently, the efficiencies of plant prime editing have also been greatly improved in a surrogate prime editing system or by modifying pegRNAs with the structured RNA motifs in the M-MLV component-engineered PPEs [13, 14]. We believe that enpPE2 and other optimized PE tools substantially extend the scope and capabilities of prime editing for use in fundamental research and molecular breeding in crops.

## Methods

### Vector construction

The plant prime editors were developed in the previously reported pHUC411-pPE2 vector [3]. To construct prime editing tools with the PEmax architecture, double R221K/N394K mutations were introduced with a site-specific mutagenesis kit (TransGen, Beijing, China). Next, the nSpCas9-R221K/N394K fragment, a synthetic NLS containing a 34 aa linker, and the M-MLV RT sequence amplified from pPE2 were sequentially assembled by using a HiFi DNA Assembly Kit (NEB, Ipswich, USA). pPEmax was established by the further addition of a c-Myc NLS to the 3′ terminus through direct PCR. In addition, a rice codon-optimized MLH1dn was synthesized (Genewiz, Suzhou, China) and assembled at the 3′ terminus of pPEmax with a P2A self-cleavage peptide sequence, resulting in the construction of pPEmax-MLHdn. For the generation of pINPE2, an artificial sequence encoding the IGFpm1-NFATC2IPp1-32 aa linker was designed in a plant codon-based manner and synthesized (Genewiz, Suzhou, China). Then, the fragment was added to the 5′ terminus of nSpCas9 in pPE2 by Gibson cloning. To construct phyPE2, the nSpCas9 and M-MLV RT sequences were amplified from pPE2, while the sequence of Rad51-ssDBD was amplified from Nm1-hyBE3 [15] and assembled between nSpCas9 and M-MLV RT. The engineered fusions were subjected to Sanger sequencing (Sangon Biotech, Shanghai, China) and then inserted immediately downstream of a maize ubiquitin 1 (ZmUBI1) promoter in a pHUC backbone by PstI/SacI double digestion [3]. An OsU3 promoter-driven cassette was integrated for pegRNA expression, generating pHUC411-pPE2max, pHUC411-pPE4max, pHUC411-pINPE2, and pHUC411-phyPE2. To generate pHUC411-enpPE2, the OsU3 promoter was replaced with a synthetic U6 composite promoter consisting of a CaMV 35S enhancer, a CmYLCV promoter, and a minimal OsU6 promoter by HindIII digestion in the pHUC411-pPE2max vector. In addition, tRNA and HDV ribozyme sequences were respectively added to the 5′ and 3′ end of pegRNA in the cassette of the U6 composite promoter by direct PCR amplification. The primers used in this study are listed in Additional file 1: Table S2.

The construction of pegRNA followed a previously described method [3]. A PBS with a 30°C Tm was designed by using a web tool (www.plantgenomeediting.net) [6]. epegRNAs were assembled with an annealed protospacer, sgRNA scaffold, RT template-PBS-8-nt linker, and structured RNA motif fragments via Golden Gate cloning. For each epegRNA, an 8-nt linker was designed at peglit.liugroup.us [9]. To express multiple epegRNAs, individual tRNA-epegRNA-HDV units were separately constructed and then combined with Golden Gate technology in the U6 composite-driven expression cassette. All pegRNAs were verified by Sanger sequencing. The sequences of pPE2 variants and the epegRNA expression cassette are shown in the Additional file 1: Supplementary Sequences.

### Rice transformation

The binary vectors were introduced into Agrobacterium strain EHA105 *via* freeze–thaw method. The PCR-positive clones were confirmed by Sanger sequencing and cultured to OD600=0.1 for callus infection. In the assays performed to examine the prime editing frequency in calli, at least three different clones of each vector were independently employed for rice transformation.

Two rice varieties (*Oryza sativa* ssp. *japonica* cv. Nipponbare and cv. Wuyungeng 31) were subjected to Agrobacterium-mediated genetic transformation following previously reported methods with some modifications [16]. Calli were induced from mature seeds for two to three weeks. Intermediate-sized, solid secondary calli were selected and incubated with Agrobacterium suspensions for 15 min. After infection, the calli were dried and allowed to recover for 5 days. For each vector, approximately 300~350 recovered calli were selected under 50 mg/L hygromycin for 4 weeks. Then, ~200 independent selection (transformation) events were employed for further regeneration. In each selection event, three to four calli were chosen and transformed for plant regeneration as an independent event under the selection pressure of 25 mg/L hygromycin. In each regeneration event, only a single plant was selected for rooting an independent line. The plant materials were grown under a 16:8 h light:dark photoperiod at 28°C.

### Sampling and genotyping

Resistant calli were sampled to determine the prime editing efficiency in stably transformed cells. After 15 days of selection, approximately 200 newly emerged calli of one transformant were collected together as a sample. Genomic DNA was extracted and purified with a plant genomic DNA isolation Kit (Tiangen Biotech, Beijing, China). Sequence-specific primers were designed to amplify the targeted region with Q5 high-fidelity polymerase. The amplicons were sequenced to generate 0.5 Gb of data per sample using the Illumina NextSeq platform according to a paired-end 150 bp (PE-150) strategy.

To genotype individual plants, 48 independent lines were randomly selected per vector. The leaves of different tillers in each line were collected to extract genomic DNA. The targeted mutations were examined in a Hi-TOM assay with a 5% threshold [17]. Some representative desired mutations and unintended byproducts were further validated by PCR- or PCR-cloning Sanger sequencing.

## Supplementary Information

**Additional file 1: Figure S1.** Schematic illustrations of pegRNA design. Figure S2. Alignments of prime editing byproducts in transgenic plants. Table S1. enpPE2-mediated prime editing across 10 additional targets in transgenic plants. Figure S3. enpPE2-mediated dual prime editing in transgenic rice. Table S2. Primers and oligos used in this study. Supplemental sequences.

**Additional file 2.** Review history.

## Data Availability

Deep sequencing data are available in the NCBI database under SRA accession numbers PRJNA816821, PRJNA816705 and PRJNA816407 [18–20].
