# Supplementary material for: Development of a highly efficient prime editor 2 system in plants
Source: Genome Biol. 2022 Jul 25;23:161. doi: 10.1186/s13059-022-02730-x (PMC9310484; doi:10.1186/s13059-022-02730-x)
Supplement: Supplementary file 1 — Additional file 1: Figure S1. Schematic illustrations of pegRNA design. Figure S2. Alignments of prime editing byproducts in transgenic plants. Table S1. enpPE2-mediated prime editing across 10 additional targets in transgenic plants. Figure S3. enpPE2-mediated dual prime editing in transgenic rice. Table S2. Primers and oligos used in this study. Supplemental sequences. [file 13059_2022_2730_MOESM1_ESM.docx]

**Additional file 1**

**Figure S1.** Schematic illustrations of pegRNA design.

**Figure S2.** Alignments of prime editing byproducts in transgenic plants.

**Table S1.** enpPE2-mediated prime editing across 10 additional targets in transgenic plants.

**Figure S3.** enpPE2-mediated dual prime editing in transgenic rice.

**Table S2.** Primers and oligos used in this study.

**Supplemental sequences**


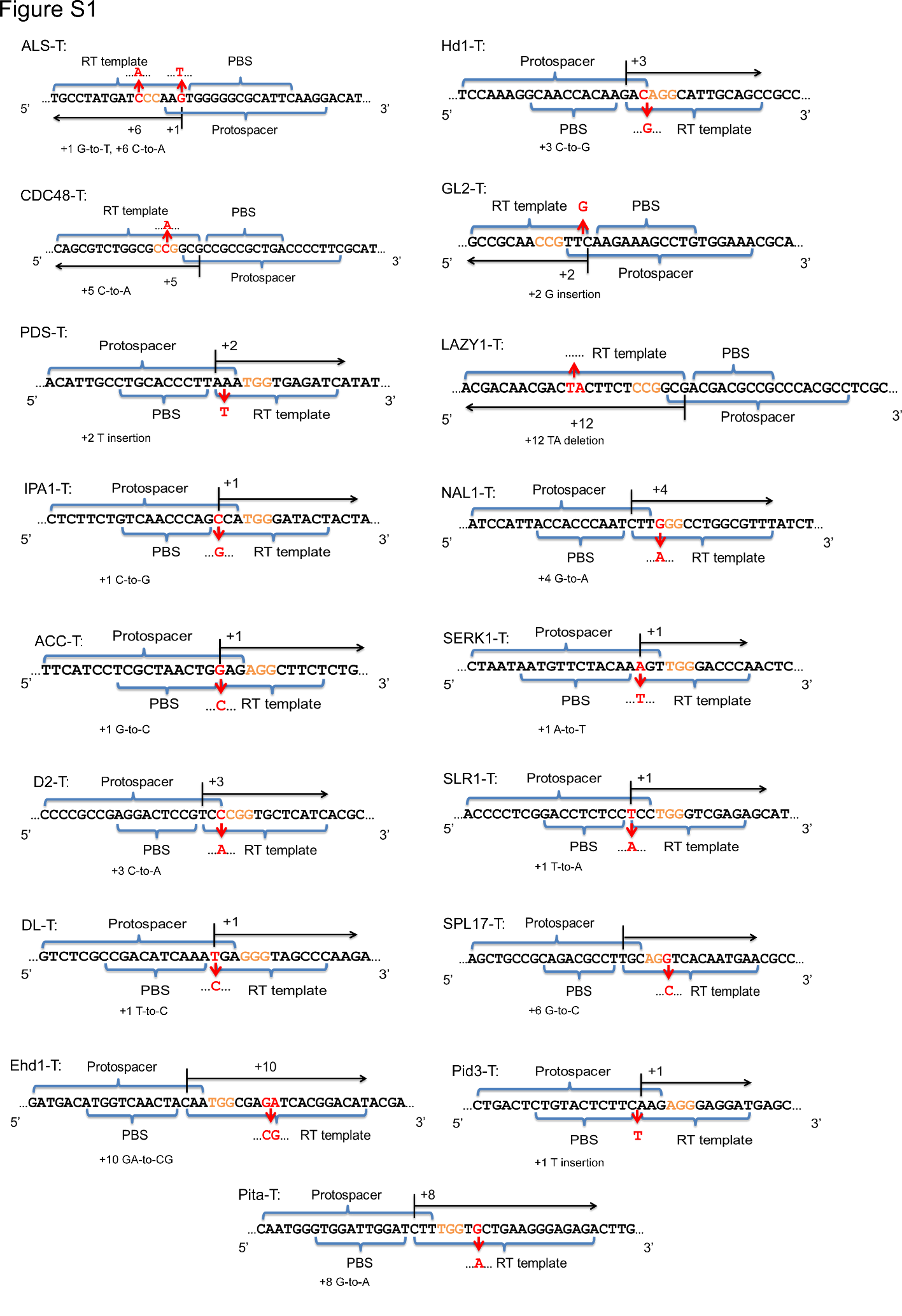


**Figure S1.** Schematic illustrations of pegRNA design.

For each site, the PAM used for gRNA design is labeled in orange. The region corresponding to the protospacer, PBS sequence and RT template of the pegRNA is indicated in the bracket. The expected mutations are shown in red. The position of the desired edit was counted from the nick site following the arrow.

**
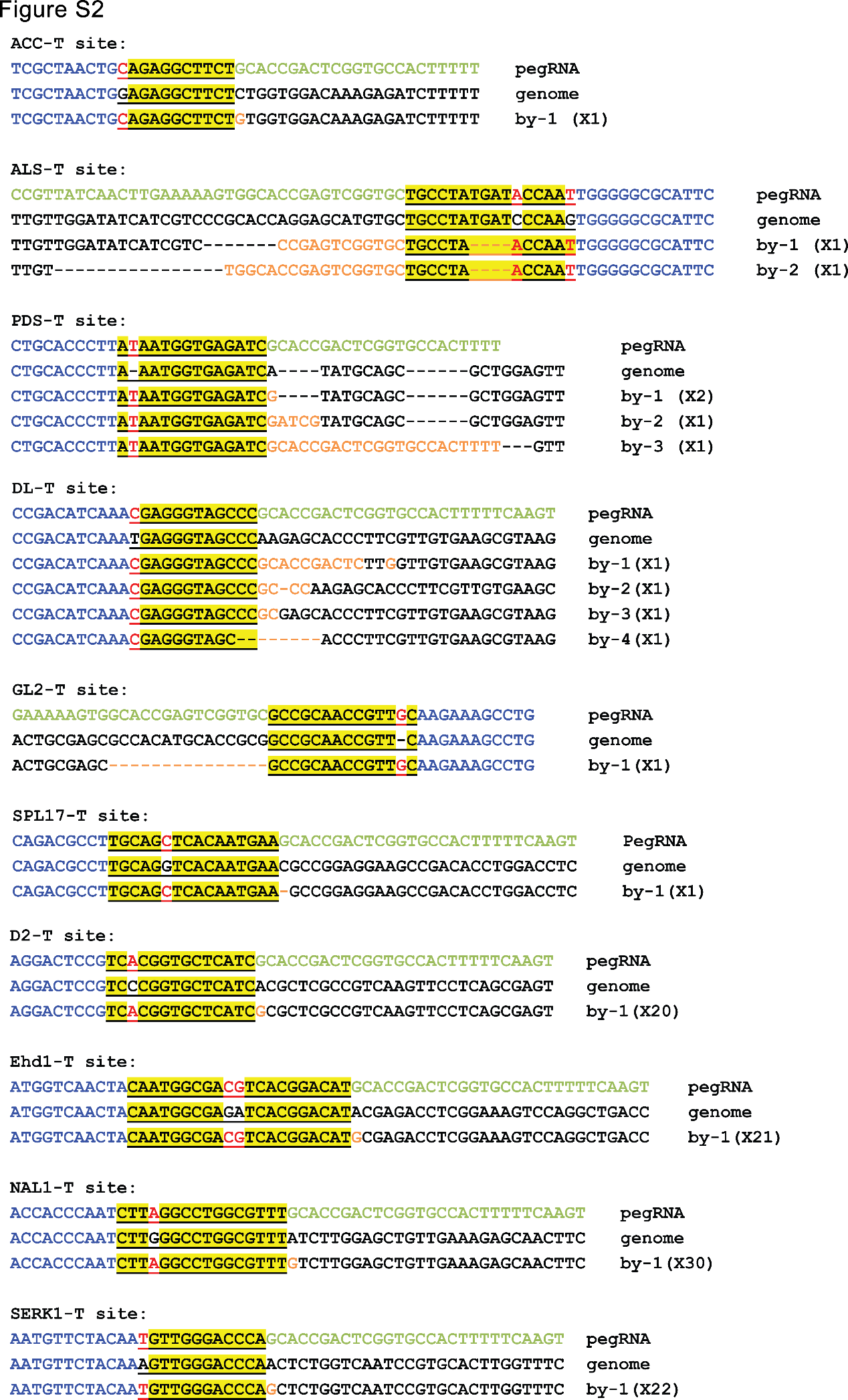
**

**Figure S2.** Alignments of prime editing byproducts in transgenic plants.

The edits were aligned with the wild-type genome sequence and pegRNA sequence. PBS is labeled in blue, and the RT template is underlined and shaded in yellow. The sgRNA scaffold sequence flanking the RT template is labeled in green. The desired mutations and unintended byproducts are labeled in red and orange, respectively. The numbers of lines carrying the byproduct mutations are indicated on the right. Note: all plants carrying high-frequency byproduct mutations at D2-T, Ehd1-T, NAL1-T and SERK1-T have a conserved A-to-G substitution at the position immediately flanking the RT template sequence.

**Table S1.** enpPE2-mediated prime editing across 10 additional targets in transgenic plants

| Targets | Mutation types* | Tested plants | Plants carrying mutation at the target position | | | Plants with byproducts^#^ |
| --- | --- | --- | --- | --- | --- | --- |
|  |  |  | Ho | He | Total (%) |  |
| D2-T | C-to-A | 48 | 19 | 12 | 31 (64.58%) | 20 |
| DL-T | T-to-C | 48 | 12 | 25 | 37 (77.08%) | 4 |
| Ehd1-T | GA-to-CG | 48 | 18 | 26 | 44 (91.67%) | 21 |
| Hd1-T | C-to-G | 48 | 7 | 11 | 18 (37.50%) | 0 |
| GL2-T | G insertion | 48 | 8 | 19 | 27 (56.25%) | 1 |
| LAZY1-T | TA deletions | 48 | 0 | 2 | 2 (4.17%) | 0 |
| NAL1-T | G-to-A | 48 | 9 | 21 | 30 (62.50%) | 30 |
| SERK1-T | A-to-T | 48 | 6 | 19 | 25 (52.08%) | 22 |
| SLR1-T | T-to-A | 48 | 0 | 7 | 7 (14.58%) | 0 |
| SPL17-T | G-to-C | 48 | 25 | 9 | 34 (70.83%) | 1 |

*, Ho, homozygous desired mutation at the target position; He, heterozygous desired mutation at the target position.

#, please note that plants with byproducts at D2-T, Ehd1-T, NAL1-T, and SERK1-T are normally chimeric/biallelic mutants simultaneously carrying the desired edit and a composite edit consisting of both the desired and byproduct mutations.


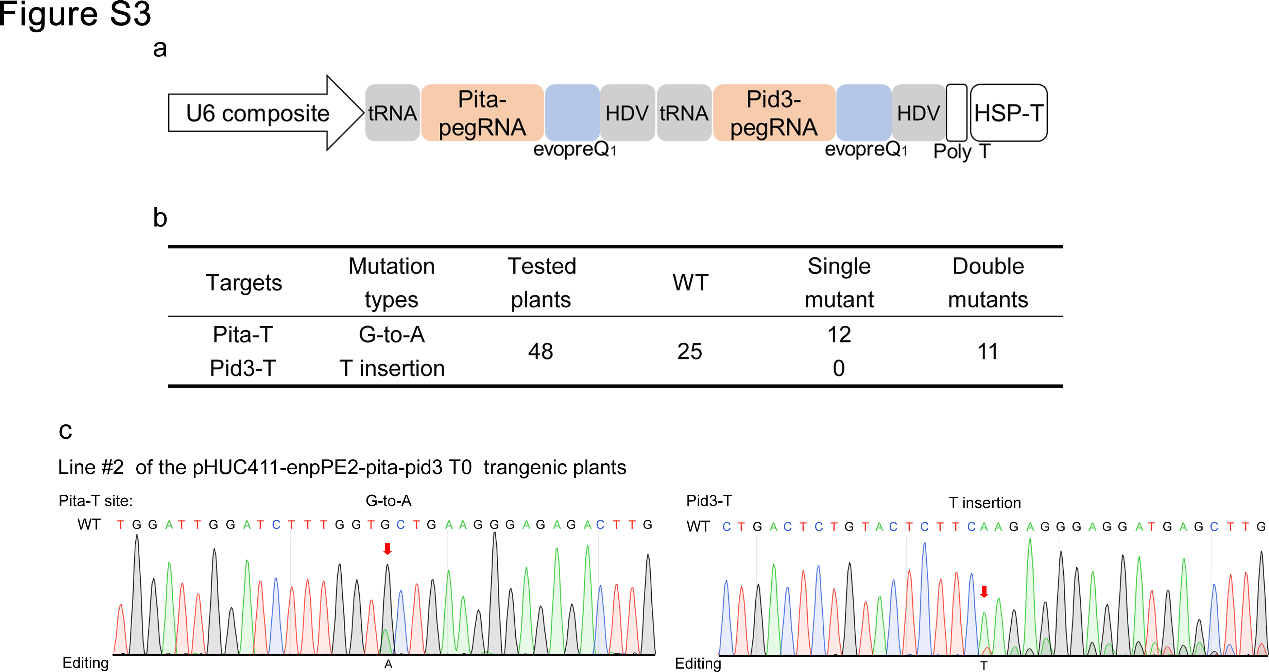


**Figure S3.** enpPE2-mediated dual prime editing in transgenic rice.

a, Schematic representation of the pegRNA expression cassette for simultaneously editing the Pita-T and Pid3-T sites in an enpPE2 vector. Poly T, poly T terminator; HSP-T, AtHSP18.2 terminator. b, Editing at the Pita-T and Pid3-T sites in transgenic plants. Mutations were identified by Hi-TOM analysis with a 5% threshold. c, Sanger sequencing of a representative T_0_ edited line at the Pita-T and Pid3-T sites.

**Table S2.** Primers and oligos used in this study.

1. pegRNA sequences used in the study

| Target | pegRNA | Sequence |
| --- | --- | --- |
| ALS-T | pegRNA | CCTTGAATGCGCCCCCACTTgtttcagagctatgctggaaacagcatagcaagttgaaataaggctagtccgttatcaacttgaaaaagtggcaccgagtcggtgcTGCCTATGATACCAATTGGGGGCGCATTC |
|  | pegRNA-mpknot | CCTTGAATGCGCCCCCACTTgtttcagagctatgctggaaacagcatagcaagttgaaataaggctagtccgttatcaacttgaaaaagtggcaccgagtcggtgcTGCCTATGATACCAATTGGGGGCGCATTCTATATACCGGGTCAGGAGCCCCCCCCCTGAACCCAGGATAACCCTCAAAGTCGGGGGGCAACCC |
|  | pegRNA-evopreQ_1_ | CCTTGAATGCGCCCCCACTTgtttcagagctatgctggaaacagcatagcaagttgaaataaggctagtccgttatcaacttgaaaaagtggcaccgagtcggtgcTGCCTATGATACCAATTGGGGGCGCATTCattcttaaTTGACGCGGTTCTATCTAGTTACGCGTTAAACCAACTAGAAA |
| CDC48-T | pegRNA | GAAGGGGTCAGCGGCGGCGCgtttcagagctatgctggaaacagcatagcaagttgaaataaggctagtccgttatcaacttgaaaaagtggcaccgagtcggtgcCAGCGTCTGGCGCAGGCGCCGCCGCTGA |
|  | pegRNA-mpknot | GAAGGGGTCAGCGGCGGCGCgtttcagagctatgctggaaacagcatagcaagttgaaataaggctagtccgttatcaacttgaaaaagtggcaccgagtcggtgcCAGCGTCTGGCGCAGGCGCCGCCGCTGAGGGAAAGAGGGTCAGGAGCCCCCCCCCTGAACCCAGGATAACCCTCAAAGTCGGGGGGCAACCC |
|  | pegRNA-evopreQ_1_ | GAAGGGGTCAGCGGCGGCGCgtttcagagctatgctggaaacagcatagcaagttgaaataaggctagtccgttatcaacttgaaaaagtggcaccgagtcggtgcCAGCGTCTGGCGCAGGCGCCGCCGCTGAggaaggacTTGACGCGGTTCTATCTAGTTACGCGTTAAACCAACTAGAAA |
| PDS-T | pegRNA | ACATTGCCTGCACCCTTAAAgtttcagagctatgctggaaacagcatagcaagttgaaataaggctagtccgttatcaacttgaaaaagtggcaccgagtcggtgcGATCTCACCATTATAAGGGTGCAG |
|  | pegRNA-mpknot | ACATTGCCTGCACCCTTAAAgtttcagagctatgctggaaacagcatagcaagttgaaataaggctagtccgttatcaacttgaaaaagtggcaccgagtcggtgcGATCTCACCATTATAAGGGTGCAGaatttctcGGGTCAGGAGCCCCCCCCCTGAACCCAGGATAACCCTCAAAGTCGGGGGGCAACCC |
|  | pegRNA-evopreQ_1_ | ACATTGCCTGCACCCTTAAAgtttcagagctatgctggaaacagcatagcaagttgaaataaggctagtccgttatcaacttgaaaaagtggcaccgagtcggtgcGATCTCACCATTATAAGGGTGCAGaactacacTTGACGCGGTTCTATCTAGTTACGCGTTAAACCAACTAGAAA |
| ACC-T | pegRNA | TTCATCCTCGCTAACTGGAGgtttcagagctatgctggaaacagcatagcaagttgaaataaggctagtccgttatcaacttgaaaaagtggcaccgagtcggtgcAGAAGCCTCTGCAGTTAGCGA |
|  | pegRNA-evopreQ_1_ | TTCATCCTCGCTAACTGGAGgtttcagagctatgctggaaacagcatagcaagttgaaataaggctagtccgttatcaacttgaaaaagtggcaccgagtcggtgcAGAAGCCTCTGCAGTTAGCGAtcccaattTTGACGCGGTTCTATCTAGTTACGCGTTAAACCAACTAGAAA |
| IPA1-T | pegRNA | CTCTTCTGTCAACCCAGCCAgtttcagagctatgctggaaacagcatagcaagttgaaataaggctagtccgttatcaacttgaaaaagtggcaccgagtcggtgcagtatcccatgCctgggttgac |
|  | pegRNA-mpknot | CTCTTCTGTCAACCCAGCCAgtttcagagctatgctggaaacagcatagcaagttgaaataaggctagtccgttatcaacttgaaaaagtggcaccgagtcggtgcagtatcccatgCctgggttgaccttacaccGGGTCAGGAGCCCCCCCCCTGAACCCAGGATAACCCTCAAAGTCGGGGGGCAACCC |
|  | pegRNA-evopreQ_1_ | CTCTTCTGTCAACCCAGCCAgtttcagagctatgctggaaacagcatagcaagttgaaataaggctagtccgttatcaacttgaaaaagtggcaccgagtcggtgcagtatcccatgCctgggttgactttacaccTTGACGCGGTTCTATCTAGTTACGCGTTAAACCAACTAGAAA |
| Hd1-T | pegRNA-evopreQ_1_ | tccaaaggcaaccacaagaCgtttcagagctatgctggaaacagcatagcaagttgaaataaggctagtccgttatcaacttgaaaaagtggcaccgagtcggtgcgctgcaatgcctCtcttgtggttgcGAACATTTTTGACGCGGTTCTATCTAGTTACGCGTTAAACCAACTAGAAA |
| D2-T | pegRNA-evopreQ_1_ | ccccgccgaggactccgtcCgtttcagagctatgctggaaacagcatagcaagttgaaataaggctagtccgttatcaacttgaaaaagtggcaccgagtcggtgcgatgagcaccgTgacggagtcctAAATATATTTGACGCGGTTCTATCTAGTTACGCGTTAAACCAACTAGAAA |
| Pita-T | pegRNA-evopreQ_1_ | caatgggtggattggatcttgtttcagagctatgctggaaacagcatagcaagttgaaataaggctagtccgttatcaacttgaaaaagtggcaccgagtcggtgctctctcccttcagTaccaaagatccaatccacAGAGCACCTTGACGCGGTTCTATCTAGTTACGCGTTAAACCAACTAGAAA |
| GL2-T | pegRNA-evopreQ_1_ | tttccacaggctttcttgaagtttcagagctatgctggaaacagcatagcaagttgaaataaggctagtccgttatcaacttgaaaaagtggcaccgagtcggtgcgccgcaaccgttGcaagaaagcctgAATACCCGTTGACGCGGTTCTATCTAGTTACGCGTTAAACCAACTAGAAA |
| LZAY1-T | pegRNA-evopreQ_1_ | ggcgtgggcggcgtcgtcgcgtttcagagctatgctggaaacagcatagcaagttgaaataaggctagtccgttatcaacttgaaaaagtggcaccgagtcggtgcacgacaacgaccttctccggcgacgacgccgAAATTAAGTTGACGCGGTTCTATCTAGTTACGCGTTAAACCAACTAGAAA |
| SLR1-T | pegRNA-evopreQ_1_ | acccctcggacctctccTccgtttcagagctatgctggaaacagcatagcaagttgaaataaggctagtccgttatcaacttgaaaaagtggcaccgagtcggtgctctcgacccaggTggagaggtcATTTAAACTTGACGCGGTTCTATCTAGTTACGCGTTAAACCAACTAGAAA |
| DL-T | pegRNA-evopreQ_1_ | gtctcgccgacatcaaaTgagtttcagagctatgctggaaacagcatagcaagttgaaataaggctagtccgttatcaacttgaaaaagtggcaccgagtcggtgcgggctaccctcGtttgatgtcggAATCCCTGTTGACGCGGTTCTATCTAGTTACGCGTTAAACCAACTAGAAA |
| NAL1-T | pegRNA-evopreQ_1_ | atccattaccacccaatcttgtttcagagctatgctggaaacagcatagcaagttgaaataaggctagtccgttatcaacttgaaaaagtggcaccgagtcggtgcaaacgccaggccTaagattgggtggtTCCTCAGCTTGACGCGGTTCTATCTAGTTACGCGTTAAACCAACTAGAAA |
| SPL17-T | pegRNA-evopreQ_1_ | AgctgccgcagacgccttgcgtttcagagctatgctggaaacagcatagcaagttgaaataaggctagtccgttatcaacttgaaaaagtggcaccgagtcggtgcttcattgtgaGctgcaaggcgtctgAAATCTTGTTGACGCGGTTCTATCTAGTTACGCGTTAAACCAACTAGAAA |
| SERK1-T | pegRNA-evopreQ_1_ | CtaataatgttctacaaAgtgtttcagagctatgctggaaacagcatagcaagttgaaataaggctagtccgttatcaacttgaaaaagtggcaccgagtcggtgctgggtcccaacAttgtagaacattTCCCGTCCTTGACGCGGTTCTATCTAGTTACGCGTTAAACCAACTAGAAA |
| Pid3-T | pegRNA-evopreQ_1_ | CtgactctgtactcttcaaggtttcagagctatgctggaaacagcatagcaagttgaaataaggctagtccgttatcaacttgaaaaagtggcaccgagtcggtgcatcctccctcttAgaagagtacagCCTAAATTTTGACGCGGTTCTATCTAGTTACGCGTTAAACCAACTAGAAA |
| Ehd1-T | pegRNA-evopreQ_1_ | gatgacatggtcaactacaagtttcagagctatgctggaaacagcatagcaagttgaaataaggctagtccgttatcaacttgaaaaagtggcaccgagtcggtgcatgtccgtgaCGtcgccattgtagttgaccatAATAAAGTTTGACGCGGTTCTATCTAGTTACGCGTTAAACCAACTAGAAA |

2. Primers for Hi-TOM analysis on the targets

| **Target** | **Sequence** |
| --- | --- |
| PDS-T | FP: ggagtgagtacggtgtgcTTGGTATTAATGATCGGTTGCA |
|  | RP: gagttggatgctggatggTACTTCTGGGAATCTTGGTTTA |
| ACC-T | FP: ggagtgagtacggtgtgcGCAGGCATTGCTGGACTTCAAC |
|  | RP: gagttggatgctggatggAATGTAGACAAAGGCAGGCTGA |
| ALS-T | FP: ggagtgagtacggtgtgcAAGAAGATGCTCGAGACTCCAG |
|  | RP: gagttggatgctggatggGCTGGTGCTTTGCCAACATACA |
| CDC48-T | FP: ggagtgagtacggtgtgcAGTCTCGTGGGTTCGGCACCGA |
|  | RP: gagttggatgctggatggTAAATATAAATAGCACAGCGAT |
| Hd1-T | FP: ggagtgagtacggtgtgcGGTGAGAAGAGAAACGCTGA |
|  | RP: gagttggatgctggatggCAAGTCGAATCCAGCGATTG |
| SLR1-T | FP: ggagtgagtacggtgtgcGGGGATGGGCGGCGTGAGCG |
|  | RP: gagttggatgctggatggTGGCGGGATAGGGGGCAGCGG |
| D2-T | FP: ggagtgagtacggtgtgccaccgacgagctcatctccg |
|  | RP: gagttggatgctggatgggcagggcgagagggcactcg |
| Pit-Ta | FP: ggagtgagtacggtgtgcGTCTGCCTTGAGGACTGCAATT |
|  | RP: gagttggatgctggatggATACCCGTGATCATCTTGTTGT |
| GL2-T | FP: ggagtgagtacggtgtgcACGGACGGCAAGAAATGGCG |
|  | RP: gagttggatgctggatggGCCGCAGAACCGACAACAGATG |
| LAZY1-T | FP: ggagtgagtacggtgtgctaATTCACTGTGTGACTGCAGG |
|  | RP: gagttggatgctggatggTGCCGCCGAAGGTGAAGAGGTC |
| DL-T | FP: ggagtgagtacggtgtgcTTCTTGGTGTTAGGGACCTTGC |
|  | RP:gagttggatgctggatggCGTGTATGATATAGCAAGTGAAGTG |
| NAL1-T | FP: ggagtgagtacggtgtgcGACTTGGACTACCCTAATCA |
|  | RP: gagttggatgctggatggAGATTCCATACCAAACGTCA |
| SPL17-T | FP: ggagtgagtacggtgtgcTTACCTGAATTTGATCAAGA |
|  | RP: gagttggatgctggatggGGATGCAGCAAGCCGGCCAT |
| SERK1-T | FP: ggagtgagtacggtgtgcGTGATGCATTGCATAGCTTG |
|  | RP: gagttggatgctggatggACTCTGATAACACTGTTGTC |
| Pid3-T | FP: ggagtgagtacggtgtgcATCGATGGCGGGAAGGAGGA |
|  | RP: gagttggatgctggatggCTTTCACCCATTTCATCAGC |
| Ehd1-T | FP: ggagtgagtacggtgtgcGAAGCTTCGCTAGCGCCTCT |
|  | RP: gagttggatgctggatggTGACGGTGCAACTGCGTGGT |

3. Primers for Sanger sequencing analysis on the targets

| **Target** | **Sequence** |
| --- | --- |
| PDS-T | FP: TCACACTGTTTTGTCGTCCACA |
|  | RP: TTCCTGTTAAATGCACGCATGA |
| ACC-T | FP: TTGATGACAGCCAAGGGAAATG |
|  | RP: ATGCGGTCTGGGTTTATCTTGC |
| CDC48-T | FP: CGGAGGAAGGACAACCCTGAAG |
|  | RP: ATACAACGCAAATCTATCCATG |
| ALS-T | FP: AACATTTGGGTATGGTGGTGCA |
|  | RP: TTGCATAGAAGTACTTTATTCT |
| Hd1-T | FP: GAATCAGTGTACTCATCACTAA |
|  | RP: CTACCTCGCATGGCTCTTGTGG |
| SLR1-T | FP: CCGATATGGGGTCGTGCAAGG |
|  | RP: CATCCGCTTGGTGTCCCTCGC |
| D2-T | FP: gatggcgaggctgatacagagg |
|  | RP: GACTGCCCATGCCCAGCAACAG |
| Pita-T | FP: ACAGGGTTGGAACACTTGGT |
|  | RP: CCGAGAAAATATAGGACCTCCC |
| GL2-T | FP: CGGTTGCTACGATGTGCCTGTT |
|  | RP: AGAACCCAACGCCGAGCCAAAT |
| LAZY1-T | FP: GATAAGACGGACGGTCAAACAT |
|  | RP: GGAAGGTGAAGGTGGGCGTGAC |
| DL-T | FP: ATGCATGGCAAGCGTTTGAGAG |
|  | RP: GTTGTAAGCAGATGGGAGGCGG |
| NAL1-T | FP: acggatggagtacttctgaagc |
|  | RP: gtagataaagccctgaccagct |
| SPL17-T | FP: ATAGCATGACATTCTTCATT |
|  | RP: TCTGGACCTGCCTGGCTCTT |
| SERK1-T | FP: CAATCTACACAACAATTATTAG |
|  | RP: TAGTTGCCCAAGTTGTGGGA |
| Pid3-T | FP: AAGGAGACGGCCGGATGGGG |
|  | RP: CTTCCGATCATTCTTGCGAA |
| Ehd1-T | FP: TACAGTGATGAAATATGTTG |
|  | RP: TCTTGTCAAATGTTTGACCTTC |

4. Primers for amplicon deep sequencing

| **Target** | **Sequence** |
| --- | --- |
| ALS-T | FP: CTCGAGACTCCAGGGCCATA |
|  | RP: TTCAGGTCAAACATAGGCCG |
| CDC48-T | FP: ATCCGCAAGTACCAGGCCTTC |
|  | RP: ACTGAACAAAAGCACGGAGGTTA |
| IPA1-T | FP: TAGCTCTTCTTCAGGGCCACC |
|  | RP: CAAAGCTGGTGGTAGTGGACAT |
| ACC-T | FP: TTCCTCGTGCTGGACAAGTG |
|  | RP: GGCAGGCTGATTGTATGTCCT |
| PDS-T | FP: CGGTTGCAATGGAAGGAACAC |
|  | RP: ACCCCCTAATTACAGTCATGTG |

**Supplemental sequences**

>pPEmax

ATGGCGccaaagaagaagcgcaaggtcgacaagaagtactccatcggcctcgacatcggcaccaattctgttggctgggccgtgatcaccgacgagtacaaggtgccgtccaagaagttcaaggtcctcggcaacaccgaccgccactccatcaagaagaatctcatcggcgccctgctgttcgactctggcgagacagccgaggctacaaggctcaagaggaccgctagacgcaggtacaccaggcgcaagaaccgcatctgctacctccaagagatcttctccaacgagatggccaaggtggacgacagcttcttccacaggctcgaggagagcttcctcgtcgaggaggacaagaagcacgagcgccatccgatcttcggcaacatcgtggatgaggtggcctaccacgagaagtacccgaccatctaccacctccgcaagaagctcgtcgactccaccgataaggccgacctcaggctcatctacctcgccctcgcccacatgatcaagttcaggggccacttcctcatcgagggcgacctcaacccggacaactccgatgtggacaagctgttcatccagctcgtgcagacctacaaccagctgttcgaggagaacccgatcaacgcctctggcgttgacgccaaggctattctctctgccaggctctctaagtcccgcaAgctcgagaatctgatcgcccaacttccgggcgagaagaagaatggcctcttcggcaacctgatcgccctctctcttggcctcaccccgaacttcaagtccaacttcgacctcgccgaggacgccaagctccagctttccaaggacacctacgacgacgacctcgacaatctcctcgcccagattggcgatcagtacgccgatctgttcctcgccgccaagaatctctccgacgccatcctcctcagcgacatcctcagggtgaacaccgagatcaccaaggccccactctccgcctccatgatcaagaggtacgacgagcaccaccaggacctcacactcctcaaggccctcgtgagacagcagctcccagagaagtacaaggagatcttcttcgaccagtccaagaacggctacgccggctacatcgatggcggcgcttctcaagaggagttctacaagttcatcaagccgatcctcgagaagatggacggcaccgaggagctgctcgtgaagctcaaGagagaggacctcctccgcaagcagcgcaccttcgataatggctccatcccgcaccagatccacctcggcgagcttcatgctatcctccgcaggcaagaggacttctacccgttcctcaaggacaaccgcgagaagattgagaagatcctcaccttccgcatcccgtactacgtgggcccgctcgccaggggcaactccaggttcgcctggatgaccagaaagtccgaggagacaatcaccccctggaacttcgaggaggtggtggataagggcgcctctgcccagtctttcatcgagcgcatgaccaacttcgacaagaacctcccgaacgagaaggtgctcccgaagcactcactcctctacgagtacttcaccgtgtacaacgagctgaccaaggtgaagtacgtgaccgaggggatgaggaagccagctttccttagcggcgagcaaaagaaggccatcgtcgacctgctgttcaagaccaaccgcaaggtgaccgtgaagcagctcaaggaggactacttcaagaaaatcgagtgcttcgactccgtcgagatctccggcgtcgaggataggttcaatgcctccctcgggacctaccacgacctcctcaagattatcaaggacaaggacttcctcgacaacgaggagaacgaggacatcctcgaggacatcgtgctcaccctcaccctcttcgaggaccgcgagatgatcgaggagcgcctcaagacatacgcccacctcttcgacgacaaggtgatgaagcagctgaagcgcaggcgctataccggctggggcaggctctctaggaagctcatcaacggcatccgcgacaagcagtccggcaagacgatcctcgacttcctcaagtccgacggcttcgccaaccgcaacttcatgcagctcatccacgacgactccctcaccttcaaggaggacatccaaaaggcccaggtgtccggccaaggcgattccctccatgaacatatcgccaatctcgccggctccccggctatcaagaagggcattctccagaccgtgaaggtggtggacgagctggtgaaggtgatgggcaggcacaagccagagaacatcgtgatcgagatggcccgcgagaaccagaccacacagaagggccaaaagaactcccgcgagcgcatgaagaggatcgaggagggcattaaggagctgggctcccagatcctcaaggagcacccagtcgagaacacccagctccagaacgagaagctctacctctactacctccagaacggccgcgacatgtacgtggaccaagagctggacatcaaccgcctctccgactacgacgtggacGCtattgtgccgcagtccttcctgaaggacgactccatcgacaacaaggtgctcacccgctccgacaagaacaggggcaagtccgataacgtgccgtccgaagaggtcgtcaagaagatgaagaactactggcgccagctcctcaacgccaagctcatcacccagaggaagttcgacaacctcaccaaggccgagagaggcggcctttccgagcttgataaggccggcttcatcaagcgccagctcgtcgagacacgccagatcacaaagcacgtggcccagatcctcgactcccgcatgaacaccaagtacgacgagaacgacaagctcatccgcgaggtgaaggtcatcaccctcaagtccaagctcgtgtccgacttccgcaaggacttccagttctacaaggtgcgcgagatcaacaactaccaccacgcccacgacgcctacctcaatgccgtggtgggcacagccctcatcaagaagtacccaaagctcgagtccgagttcgtgtacggcgactacaaggtgtacgacgtgcgcaagatgatcgccaagtccgagcaagagatcggcaaggcgaccgccaagtacttcttctactccaacatcatgaatttcttcaagaccgagatcacgctcgccaacggcgagattaggaagaggccgctcatcgagacaaacggcgagacaggcgagatcgtgtgggacaagggcagggatttcgccacagtgcgcaaggtgctctccatgccgcaagtgaacatcgtgaagaagaccgaggttcagaccggcggcttctccaaggagtccatcctcccaaagcgcaactccgacaagctgatcgcccgcaagaaggactgggacccgaagaagtatggcggcttcgattctccgaccgtggcctactctgtgctcgtggttgccaaggtcgagaagggcaagagcaagaagctcaagtccgtcaaggagctgctgggcatcacgatcatggagcgcagcagcttcgagaagaacccaatcgacttcctcgaggccaagggctacaaggaggtgaagaaggacctcatcatcaagctcccgaagtacagcctcttcgagcttgagaacggccgcaagagaatgctcgcctctgctggcgagcttcagaagggcaacgagcttgctctcccgtccaagtacgtgaacttcctctacctcgcctcccactacgagaagctcaagggctccccagaggacaacgagcaaaagcagctgttcgtcgagcagcacaagcactacctcgacgagatcatcgagcagatctccgagttctccaagcgcgtgatcctcgccgatgccaacctcgataaggtgctcagcgcctacaacaagcaccgcgataagccaattcgcgagcaggccgagaacatcatccacctcttcaccctcaccaacctcggcgctccagccgccttcaagtacttcgacaccaccatcgaccgcaagcgctacacctctaccaaggaggttctcgacgccaccctcatccaccagtctatcacaggcctctacgagacacgcatcgacctctcacaactcggcggcgatTCAGGCGGCTCCAGCGGCGGCTCTAAGCGGACCGCCGACGGATCAGAGTTCGAGAGCCCGAAGAAGAAGAGGAAGGTGTCCGGCGGCTCATCTGGCGGCTCCACACTCAATATCGAGGACGAGTACAGGCTGCATGAGACATCCAAGGAGCCTGACGTCTCCCTCGGCAGCACATGGCTCTCAGATTTCCCACAGGCCTGGGCCGAGACAGGCGGCATGGGCCTCGCCGTCCGCCAGGCGCCGCTCATCATTCCACTGAAGGCGACCTCCACACCGGTGAGCATCAAGCAGTACCCAATGTCTCAGGAGGCAAGGCTGGGCATCAAGCCACACATTCAGAGGCTCCTGGACCAGGGCATTCTGGTGCCTTGCCAGAGCCCGTGGAACACCCCTCTCCTGCCGGTGAAGAAGCCTGGCACAAATGACTACCGCCCGGTCCAGGATCTCAGGGAGGTGAACAAGCGCGTCGAGGATATCCATCCGACAGTCCCGAACCCATACAATCTCCTGTCAGGCCTCCCGCCATCTCACCAGTGGTACACCGTGCTCGACCTGAAGGATGCGTTCTTCTGCCTCAGGCTGCATCCAACAAGCCAGCCTCTCTTCGCCTTCGAGTGGCGCGATCCGGAGATGGGCATTTCAGGCCAGCTCACCTGGACACGGCTGCCACAGGGCTTCAAGAACTCTCCTACCCTCTTCAATGAGGCGCTCCATCGGGACCTGGCCGATTTCAGGATCCAGCACCCAGACCTCATTCTCCTCCAGTATGTGGACGATCTCCTGCTCGCCGCGACATCCGAGCTGGATTGCCAGCAGGGAACCCGCGCGCTGCTCCAGACACTGGGAAATCTGGGATACAGGGCATCAGCGAAGAAGGCACAGATCTGCCAGAAGCAGGTCAAGTACCTCGGCTACCTGCTCAAGGAGGGACAGAGGTGGCTGACAGAGGCAAGGAAGGAGACAGTGATGGGCCAGCCTACCCCGAAGACACCACGGCAGCTCAGGGAGTTCCTGGGCAAGGCGGGCTTCTGCCGCCTCTTCATCCCAGGATTCGCGGAGATGGCGGCGCCACTCTACCCTCTGACCAAGCCTGGCACACTGTTCAACTGGGGACCAGACCAGCAGAAGGCGTACCAGGAGATTAAGCAGGCCCTGCTCACAGCACCTGCCCTCGGCCTGCCGGACCTCACAAAGCCATTCGAGCTGTTCGTGGATGAGAAGCAGGGCTACGCGAAGGGAGTCCTGACACAGAAGCTGGGACCATGGAGGCGCCCAGTGGCCTACCTCTCCAAGAAGCTGGACCCAGTGGCTGCCGGCTGGCCTCCGTGCCTGAGGATGGTGGCGGCCATTGCCGTCCTCACCAAGGATGCCGGCAAGCTGACAATGGGCCAGCCTCTCGTCATTCTGGCGCCGCATGCGGTGGAGGCGCTCGTCAAGCAGCCACCTGATAGGTGGCTGTCCAACGCGCGCATGACCCACTACCAGGCCCTGCTCCTGGACACAGATAGGGTGCAGTTCGGCCCAGTGGTCGCCCTCAATCCTGCCACACTGCTGCCACTCCCTGAGGAGGGCCTCCAGCATAACTGCCTCGATATTCTGGCGGAGGCCCATGGAACCCGCCCTGACCTCACAGATCAGCCGCTGCCAGACGCCGATCACACCTGGTACACAGATGGCTCATCTCTCCTCCAGGAGGGCCAGAGGAAGGCCGGAGCCGCGGTGACCACAGAGACAGAGGTCATCTGGGCAAAGGCGCTCCCAGCCGGCACCTCCGCACAGAGGGCCGAGCTGATTGCACTGACACAGGCGCTCAAGATGGCCGAGGGCAAGAAGCTGAATGTGTACACCGACTCACGCTACGCCTTCGCGACAGCCCACATCCATGGAGAGATCTACAGGAGGAGGGGATGGCTCACATCTGAGGGCAAGGAGATCAAGAACAAGGATGAGATTCTCGCGCTCCTGAAGGCCCTCTTCCTGCCAAAGCGCCTGTCAATCATTCACTGCCCTGGCCATCAGAAGGGACACTCTGCGGAGGCAAGGGGAAATAGGATGGCCGACCAGGCGGCCAGGAAGGCAGCGATCACCGAGACACCGGATACCTCCACACTCCTGATTGAGAACTCCAGCCCATCAGGCGGCTCTAAGAGGACCGCCGACGGATCAGAGTTCGAGAGCCCGAAGAAGAAGAGGAAAGTGGGATCAGGACCAGCCGCCAAGAGGGTGAAGCTCGATTGA

Black, nSpCas9-R221K/N394K/H840A;

Blue, SV40 NLS;

Yellow, c-Myc NLS;

Green, NLS-embedded 34aa liner;

Orange, M-MLV RT.

>pPEmax-MLH1dn

atggccccaaagaagaagcgcaaggtcgacaagaagtactccatcggcctcgacatcggcaccaattctgttggctgggccgtgatcaccgacgagtacaaggtgccgtccaagaagttcaaggtcctcggcaacaccgaccgccactccatcaagaagaatctcatcggcgccctgctgttcgactctggcgagacagccgaggctacaaggctcaagaggaccgctagacgcaggtacaccaggcgcaagaaccgcatctgctacctccaagagatcttctccaacgagatggccaaggtggacgacagcttcttccacaggctcgaggagagcttcctcgtcgaggaggacaagaagcacgagcgccatccgatcttcggcaacatcgtggatgaggtggcctaccacgagaagtacccgaccatctaccacctccgcaagaagctcgtcgactccaccgataaggccgacctcaggctcatctacctcgccctcgcccacatgatcaagttcaggggccacttcctcatcgagggcgacctcaacccggacaactccgatgtggacaagctgttcatccagctcgtgcagacctacaaccagctgttcgaggagaacccgatcaacgcctctggcgttgacgccaaggctattctctctgccaggctctctaagtcccgcaagctcgagaatctgatcgcccaacttccgggcgagaagaagaatggcctcttcggcaacctgatcgccctctctcttggcctcaccccgaacttcaagtccaacttcgacctcgccgaggacgccaagctccagctttccaaggacacctacgacgacgacctcgacaatctcctcgcccagattggcgatcagtacgccgatctgttcctcgccgccaagaatctctccgacgccatcctcctcagcgacatcctcagggtgaacaccgagatcaccaaggccccactctccgcctccatgatcaagaggtacgacgagcaccaccaggacctcacactcctcaaggccctcgtgagacagcagctcccagagaagtacaaggagatcttcttcgaccagtccaagaacggctacgccggctacatcgatggcggcgcttctcaagaggagttctacaagttcatcaagccgatcctcgagaagatggacggcaccgaggagctgctcgtgaagctcaaGagagaggacctcctccgcaagcagcgcaccttcgataatggctccatcccgcaccagatccacctcggcgagcttcatgctatcctccgcaggcaagaggacttctacccgttcctcaaggacaaccgcgagaagattgagaagatcctcaccttccgcatcccgtactacgtgggcccgctcgccaggggcaactccaggttcgcctggatgaccagaaagtccgaggagacaatcaccccctggaacttcgaggaggtggtggataagggcgcctctgcccagtctttcatcgagcgcatgaccaacttcgacaagaacctcccgaacgagaaggtgctcccgaagcactcactcctctacgagtacttcaccgtgtacaacgagctgaccaaggtgaagtacgtgaccgaggggatgaggaagccagctttccttagcggcgagcaaaagaaggccatcgtcgacctgctgttcaagaccaaccgcaaggtgaccgtgaagcagctcaaggaggactacttcaagaaaatcgagtgcttcgactccgtcgagatctccggcgtcgaggataggttcaatgcctccctcgggacctaccacgacctcctcaagattatcaaggacaaggacttcctcgacaacgaggagaacgaggacatcctcgaggacatcgtgctcaccctcaccctcttcgaggaccgcgagatgatcgaggagcgcctcaagacatacgcccacctcttcgacgacaaggtgatgaagcagctgaagcgcaggcgctataccggctggggcaggctctctaggaagctcatcaacggcatccgcgacaagcagtccggcaagacgatcctcgacttcctcaagtccgacggcttcgccaaccgcaacttcatgcagctcatccacgacgactccctcaccttcaaggaggacatccaaaaggcccaggtgtccggccaaggcgattccctccatgaacatatcgccaatctcgccggctccccggctatcaagaagggcattctccagaccgtgaaggtggtggacgagctggtgaaggtgatgggcaggcacaagccagagaacatcgtgatcgagatggcccgcgagaaccagaccacacagaagggccaaaagaactcccgcgagcgcatgaagaggatcgaggagggcattaaggagctgggctcccagatcctcaaggagcacccagtcgagaacacccagctccagaacgagaagctctacctctactacctccagaacggccgcgacatgtacgtggaccaagagctggacatcaaccgcctctccgactacgacgtggacGCtattgtgccgcagtccttcctgaaggacgactccatcgacaacaaggtgctcacccgctccgacaagaacaggggcaagtccgataacgtgccgtccgaagaggtcgtcaagaagatgaagaactactggcgccagctcctcaacgccaagctcatcacccagaggaagttcgacaacctcaccaaggccgagagaggcggcctttccgagcttgataaggccggcttcatcaagcgccagctcgtcgagacacgccagatcacaaagcacgtggcccagatcctcgactcccgcatgaacaccaagtacgacgagaacgacaagctcatccgcgaggtgaaggtcatcaccctcaagtccaagctcgtgtccgacttccgcaaggacttccagttctacaaggtgcgcgagatcaacaactaccaccacgcccacgacgcctacctcaatgccgtggtgggcacagccctcatcaagaagtacccaaagctcgagtccgagttcgtgtacggcgactacaaggtgtacgacgtgcgcaagatgatcgccaagtccgagcaagagatcggcaaggcgaccgccaagtacttcttctactccaacatcatgaatttcttcaagaccgagatcacgctcgccaacggcgagattaggaagaggccgctcatcgagacaaacggcgagacaggcgagatcgtgtgggacaagggcagggatttcgccacagtgcgcaaggtgctctccatgccgcaagtgaacatcgtgaagaagaccgaggttcagaccggcggcttctccaaggagtccatcctcccaaagcgcaactccgacaagctgatcgcccgcaagaaggactgggacccgaagaagtatggcggcttcgattctccgaccgtggcctactctgtgctcgtggttgccaaggtcgagaagggcaagagcaagaagctcaagtccgtcaaggagctgctgggcatcacgatcatggagcgcagcagcttcgagaagaacccaatcgacttcctcgaggccaagggctacaaggaggtgaagaaggacctcatcatcaagctcccgaagtacagcctcttcgagcttgagaacggccgcaagagaatgctcgcctctgctggcgagcttcagaagggcaacgagcttgctctcccgtccaagtacgtgaacttcctctacctcgcctcccactacgagaagctcaagggctccccagaggacaacgagcaaaagcagctgttcgtcgagcagcacaagcactacctcgacgagatcatcgagcagatctccgagttctccaagcgcgtgatcctcgccgatgccaacctcgataaggtgctcagcgcctacaacaagcaccgcgataagccaattcgcgagcaggccgagaacatcatccacctcttcaccctcaccaacctcggcgctccagccgccttcaagtacttcgacaccaccatcgaccgcaagcgctacacctctaccaaggaggttctcgacgccaccctcatccaccagtctatcacaggcctctacgagacacgcatcgacctctcacaactcggcggcgatTCAGGCGGCTCCAGCGGCGGCTCTAAGCGGACCGCCGACGGATCAGAGTTCGAGAGCCCGAAGAAGAAGAGGAAGGTGTCCGGCGGCTCATCTGGCGGCTCCACACTCAATATCGAGGACGAGTACAGGCTGCATGAGACATCCAAGGAGCCTGACGTCTCCCTCGGCAGCACATGGCTCTCAGATTTCCCACAGGCCTGGGCCGAGACAGGCGGCATGGGCCTCGCCGTCCGCCAGGCGCCGCTCATCATTCCACTGAAGGCGACCTCCACACCGGTGAGCATCAAGCAGTACCCAATGTCTCAGGAGGCAAGGCTGGGCATCAAGCCACACATTCAGAGGCTCCTGGACCAGGGCATTCTGGTGCCTTGCCAGAGCCCGTGGAACACCCCTCTCCTGCCGGTGAAGAAGCCTGGCACAAATGACTACCGCCCGGTCCAGGATCTCAGGGAGGTGAACAAGCGCGTCGAGGATATCCATCCGACAGTCCCGAACCCATACAATCTCCTGTCAGGCCTCCCGCCATCTCACCAGTGGTACACCGTGCTCGACCTGAAGGATGCGTTCTTCTGCCTCAGGCTGCATCCAACAAGCCAGCCTCTCTTCGCCTTCGAGTGGCGCGATCCGGAGATGGGCATTTCAGGCCAGCTCACCTGGACACGGCTGCCACAGGGCTTCAAGAACTCTCCTACCCTCTTCAATGAGGCGCTCCATCGGGACCTGGCCGATTTCAGGATCCAGCACCCAGACCTCATTCTCCTCCAGTATGTGGACGATCTCCTGCTCGCCGCGACATCCGAGCTGGATTGCCAGCAGGGAACCCGCGCGCTGCTCCAGACACTGGGAAATCTGGGATACAGGGCATCAGCGAAGAAGGCACAGATCTGCCAGAAGCAGGTCAAGTACCTCGGCTACCTGCTCAAGGAGGGACAGAGGTGGCTGACAGAGGCAAGGAAGGAGACAGTGATGGGCCAGCCTACCCCGAAGACACCACGGCAGCTCAGGGAGTTCCTGGGCAAGGCGGGCTTCTGCCGCCTCTTCATCCCAGGATTCGCGGAGATGGCGGCGCCACTCTACCCTCTGACCAAGCCTGGCACACTGTTCAACTGGGGACCAGACCAGCAGAAGGCGTACCAGGAGATTAAGCAGGCCCTGCTCACAGCACCTGCCCTCGGCCTGCCGGACCTCACAAAGCCATTCGAGCTGTTCGTGGATGAGAAGCAGGGCTACGCGAAGGGAGTCCTGACACAGAAGCTGGGACCATGGAGGCGCCCAGTGGCCTACCTCTCCAAGAAGCTGGACCCAGTGGCTGCCGGCTGGCCTCCGTGCCTGAGGATGGTGGCGGCCATTGCCGTCCTCACCAAGGATGCCGGCAAGCTGACAATGGGCCAGCCTCTCGTCATTCTGGCGCCGCATGCGGTGGAGGCGCTCGTCAAGCAGCCACCTGATAGGTGGCTGTCCAACGCGCGCATGACCCACTACCAGGCCCTGCTCCTGGACACAGATAGGGTGCAGTTCGGCCCAGTGGTCGCCCTCAATCCTGCCACACTGCTGCCACTCCCTGAGGAGGGCCTCCAGCATAACTGCCTCGATATTCTGGCGGAGGCCCATGGAACCCGCCCTGACCTCACAGATCAGCCGCTGCCAGACGCCGATCACACCTGGTACACAGATGGCTCATCTCTCCTCCAGGAGGGCCAGAGGAAGGCCGGAGCCGCGGTGACCACAGAGACAGAGGTCATCTGGGCAAAGGCGCTCCCAGCCGGCACCTCCGCACAGAGGGCCGAGCTGATTGCACTGACACAGGCGCTCAAGATGGCCGAGGGCAAGAAGCTGAATGTGTACACCGACTCACGCTACGCCTTCGCGACAGCCCACATCCATGGAGAGATCTACAGGAGGAGGGGATGGCTCACATCTGAGGGCAAGGAGATCAAGAACAAGGATGAGATTCTCGCGCTCCTGAAGGCCCTCTTCCTGCCAAAGCGCCTGTCAATCATTCACTGCCCTGGCCATCAGAAGGGACACTCTGCGGAGGCAAGGGGAAATAGGATGGCCGACCAGGCGGCCAGGAAGGCAGCGATCACCGAGACACCGGATACCTCCACACTCCTGATTGAGAACTCCAGCCCATCAGGCGGCTCTAAGAGGACCGCCGACGGATCAGAGTTCGAGAGCCCGAAGAAGAAGAGGAAAGTGGGATCAGGACCAGCCGCCAAGAGGGTGAAGCTCGATGGATCAGGAGCAACAAACTTCAGCCTCCTGAAGCAGGCCGGAGATGTGGAGGAGAATCCAGGACCATCCTTCGTGGCGGGAGTGATCAGGCGCCTCGACGAGACAGTGGTCAACCGCATTGCTGCCGGCGAAGTGATTCAGCGGCCGGCAAACGCGATCAAGGAGATGATTGAGAATTGCCTGGACGCGAAGAGCACATCAATCCAAGTGATTGTCAAGGAGGGCGGCCTCAAGCTGATCCAGATTCAGGATAATGGCACCGGCATCCGCAAGGAGGACCTCGATATTGTCTGCGAGCGGTTCACCACATCAAAGCTCCAGTCCTTCGAGGACCTCGCCTCTATCTCCACATACGGATTCAGGGGCGAGGCCCTGGCCAGCATCTCACACGTCGCCCATGTGACCATTACCACAAAGACAGCGGACGGAAAGTGCGCATACAGGGCGTCTTACTCCGATGGCAAGCTCAAGGCCCCGCCAAAGCCTTGCGCCGGAAACCAGGGCACCCAGATCACAGTGGAGGATCTGTTCTACAACATTGCCACCCGGAGGAAGGCGCTCAAGAATCCGTCTGAGGAGTACGGCAAGATCCTGGAGGTGGTCGGCCGGTACTCCGTCCATAACGCCGGCATTAGCTTCTCAGTGAAGAAGCAGGGCGAGACAGTCGCCGACGTGAGGACACTCCCAAACGCCTCCACCGTCGATAATATCAGGTCTATTTTCGGCAATGCGGTGTCCCGCGAGCTGATCGAGATTGGCTGCGAGGACAAGACCCTCGCCTTCAAGATGAACGGCTACATCTCCAACGCGAATTACAGCGTGAAGAAGTGCATCTTCCTCCTGTTCATTAATCACCGGCTGGTCGAGTCTACATCCCTCAGGAAGGCCATCGAGACAGTGTACGCCGCGTACCTCCCAAAGAACACACATCCTTTCCTCTACCTCTCCCTGGAGATTAGCCCGCAGAACGTGGATGTCAATGTGCACCCAACCAAGCACGAGGTCCATTTCCTGCACGAGGAGTCCATCCTGGAGAGGGTGCAGCAGCATATTGAGTCAAAGCTCCTGGGCTCTAATTCCAGCCGCATGTACTTCACCCAGACACTGCTCCCAGGCCTCGCGGGACCATCAGGAGAGATGGTGAAGTCTACCACATCCCTGACCTCATCTTCCACATCAGGCAGCTCAGATAAGGTCTACGCCCACCAGATGGTCAGGACCGACAGCCGCGAGCAGAAGCTCGACGCCTTCCTCCAGCCGCTCTCAAAGCCACTGTCTTCCCAGCCGCAGGCCATCGTCACCGAGGACAAGACAGATATTAGCTCAGGCCGCGCGCGGCAGCAGGATGAGGAGATGCTGGAGCTGCCTGCACCGGCGGAGGTGGCAGCGAAGAACCAGTCCCTGGAGGGCGATACCACAAAGGGCACAAGCGAGATGTCAGAGAAGCGCGGCCCTACCTCTTCCAATCCGAGGAAGCGCCATCGGGAGGACTCTGATGTCGAGATGGTGGAGGACGATTCCCGGAAGGAGATGACCGCCGCGTGCACACCACGCAGGAGGATCATTAACCTCACATCTGTCCTGTCCCTCCAGGAGGAGATCAATGAGCAGGGCCATGAGGTGCTGAGGGAGATGCTCCACAACCATTCCTTCGTCGGCTGCGTGAATCCACAGTGGGCACTGGCCCAGCACCAGACCAAGCTCTACCTCCTGAACACCACAAAGCTGAGCGAGGAGCTGTTCTACCAGATCCTCATTTACGACTTCGCCAATTTCGGAGTCCTGAGGCTCTCAGAGCCAGCGCCTCTGTTCGACCTCGCCATGCTGGCGCTCGATAGCCCAGAGTCAGGCTGGACAGAGGAGGATGGCCCTAAGGAGGGCCTGGCCGAGTACATCGTGGAGTTCCTCAAGAAGAAGGCCGAGATGCTGGCGGACTACTTCTCCCTGGAGATTGATGAGGAGGGCAACCTGATCGGCCTCCCGCTCCTGATTGACAATTACGTCCCTCCGCTGGAGGGCCTCCCAATCTTCATTCTGCGGCTCGCCACCGAGGTGAACTGGGATGAGGAGAAGGAGTGCTTCGAGAGCCTCTCAAAGGAGTGCGCGATGTTCTACTCTATCCGCAAGCAGTACATTTCTGAGGAGTCCACACTGTCAGGACAGCAGTCAGAGGTGCCTGGATCAATCCCGAACTCCTGGAAGTGGACCGTCGAGCACATCGTGTACAAGGCCCTGAGGAGCCATATTCTCCCACCTAAGCACTTCACCGAGGACGGCAACATCCTCCAGCTCGCGAATCTGCCAGATCTCTACAAGGTCTTTCCGAAGAAGAAGAGGAAGGTGTGA

Black, nSpCas9-R221K/N394K/H840A;

Blue, SV40 NLS;

Yellow, c-Myc NLS;

Green, NLS-embedded 34aa linker;

Orange, M-MLV RT;

Purple, P2A;

Red, MLH1dn.

>pINPE2

ATGGCGCCCAAGAAGAAAAGGAAGGTGAGGGAAGGCATGAACAAACCTGAAGGATATGGTGAAGAGGAGAGAAGAGCACCACAAGAGGGCATTGTTGATGAATGCTGCTTCAGGGAATGTGATCTCCGGCGACTAGAGATGTACTGCGCGCCGCTCAAGCCGGCCAAGGAAGCACGGGAGGTTCGCGCACAGCGCCACGAGGACATGCCAAAAGAGCAGAAGTACCAGCCGCCGGAGGAAAATAAGAACGAGAAGGAGCAAAGGAGAAAAGGGGAAGAATTTGGTAGTACTGGTGGGACAGGGAGCGCGGAGCCCGTAGGAAAACGGGGCCGGTGGTCTGGGGGCAGTGGAGCCGGCAGGGGAGGCCGCGGCGGATGGGGCGGTCGTGGGCGCCGCCCCCGCGCTCAGAGGTCCCCGTCGCGTGGTACATTGGATGTGGTGTCCGTCGACCTGGTGACCGACTCTGATGAGGAGATCCTTGAAGTCGCCACCGCGCGAGGCGCGGCCGACGAGGTGGAGGTCGAGCCACCAGAACCTCCTGGCCCTGTTGCTTCAAGAGATTCCTCCGGTGGATCATCTGGTGGCTCCTCCGGCTCAGAGACGCCAGGGACGTCGGAGTCCGCTACTCCTGAGAGCAGCGGCGGCTCCTCCGGGGGATCAgacaagaagtactccatcggcctcgacatcggcaccaattctgttggctgggccgtgatcaccgacgagtacaaggtgccgtccaagaagttcaaggtcctcggcaacaccgaccgccactccatcaagaagaatctcatcggcgccctgctgttcgactctggcgagacagccgaggctacaaggctcaagaggaccgctagacgcaggtacaccaggcgcaagaaccgcatctgctacctccaagagatcttctccaacgagatggccaaggtggacgacagcttcttccacaggctcgaggagagcttcctcgtcgaggaggacaagaagcacgagcgccatccgatcttcggcaacatcgtggatgaggtggcctaccacgagaagtacccgaccatctaccacctccgcaagaagctcgtcgactccaccgataaggccgacctcaggctcatctacctcgccctcgcccacatgatcaagttcaggggccacttcctcatcgagggcgacctcaacccggacaactccgatgtggacaagctgttcatccagctcgtgcagacctacaaccagctgttcgaggagaacccgatcaacgcctctggcgttgacgccaaggctattctctctgccaggctctctaagtcccgcaggctcgagaatctgatcgcccaacttccgggcgagaagaagaatggcctcttcggcaacctgatcgccctctctcttggcctcaccccgaacttcaagtccaacttcgacctcgccgaggacgccaagctccagctttccaaggacacctacgacgacgacctcgacaatctcctcgcccagattggcgatcagtacgccgatctgttcctcgccgccaagaatctctccgacgccatcctcctcagcgacatcctcagggtgaacaccgagatcaccaaggccccactctccgcctccatgatcaagaggtacgacgagcaccaccaggacctcacactcctcaaggccctcgtgagacagcagctcccagagaagtacaaggagatcttcttcgaccagtccaagaacggctacgccggctacatcgatggcggcgcttctcaagaggagttctacaagttcatcaagccgatcctcgagaagatggacggcaccgaggagctgctcgtgaagctcaatagagaggacctcctccgcaagcagcgcaccttcgataatggctccatcccgcaccagatccacctcggcgagcttcatgctatcctccgcaggcaagaggacttctacccgttcctcaaggacaaccgcgagaagattgagaagatcctcaccttccgcatcccgtactacgtgggcccgctcgccaggggcaactccaggttcgcctggatgaccagaaagtccgaggagacaatcaccccctggaacttcgaggaggtggtggataagggcgcctctgcccagtctttcatcgagcgcatgaccaacttcgacaagaacctcccgaacgagaaggtgctcccgaagcactcactcctctacgagtacttcaccgtgtacaacgagctgaccaaggtgaagtacgtgaccgaggggatgaggaagccagctttccttagcggcgagcaaaagaaggccatcgtcgacctgctgttcaagaccaaccgcaaggtgaccgtgaagcagctcaaggaggactacttcaagaaaatcgagtgcttcgactccgtcgagatctccggcgtcgaggataggttcaatgcctccctcgggacctaccacgacctcctcaagattatcaaggacaaggacttcctcgacaacgaggagaacgaggacatcctcgaggacatcgtgctcaccctcaccctcttcgaggaccgcgagatgatcgaggagcgcctcaagacatacgcccacctcttcgacgacaaggtgatgaagcagctgaagcgcaggcgctataccggctggggcaggctctctaggaagctcatcaacggcatccgcgacaagcagtccggcaagacgatcctcgacttcctcaagtccgacggcttcgccaaccgcaacttcatgcagctcatccacgacgactccctcaccttcaaggaggacatccaaaaggcccaggtgtccggccaaggcgattccctccatgaacatatcgccaatctcgccggctccccggctatcaagaagggcattctccagaccgtgaaggtggtggacgagctggtgaaggtgatgggcaggcacaagccagagaacatcgtgatcgagatggcccgcgagaaccagaccacacagaagggccaaaagaactcccgcgagcgcatgaagaggatcgaggagggcattaaggagctgggctcccagatcctcaaggagcacccagtcgagaacacccagctccagaacgagaagctctacctctactacctccagaacggccgcgacatgtacgtggaccaagagctggacatcaaccgcctctccgactacgacgtggacGCtattgtgccgcagtccttcctgaaggacgactccatcgacaacaaggtgctcacccgctccgacaagaacaggggcaagtccgataacgtgccgtccgaagaggtcgtcaagaagatgaagaactactggcgccagctcctcaacgccaagctcatcacccagaggaagttcgacaacctcaccaaggccgagagaggcggcctttccgagcttgataaggccggcttcatcaagcgccagctcgtcgagacacgccagatcacaaagcacgtggcccagatcctcgactcccgcatgaacaccaagtacgacgagaacgacaagctcatccgcgaggtgaaggtcatcaccctcaagtccaagctcgtgtccgacttccgcaaggacttccagttctacaaggtgcgcgagatcaacaactaccaccacgcccacgacgcctacctcaatgccgtggtgggcacagccctcatcaagaagtacccaaagctcgagtccgagttcgtgtacggcgactacaaggtgtacgacgtgcgcaagatgatcgccaagtccgagcaagagatcggcaaggcgaccgccaagtacttcttctactccaacatcatgaatttcttcaagaccgagatcacgctcgccaacggcgagattaggaagaggccgctcatcgagacaaacggcgagacaggcgagatcgtgtgggacaagggcagggatttcgccacagtgcgcaaggtgctctccatgccgcaagtgaacatcgtgaagaagaccgaggttcagaccggcggcttctccaaggagtccatcctcccaaagcgcaactccgacaagctgatcgcccgcaagaaggactgggacccgaagaagtatggcggcttcgattctccgaccgtggcctactctgtgctcgtggttgccaaggtcgagaagggcaagagcaagaagctcaagtccgtcaaggagctgctgggcatcacgatcatggagcgcagcagcttcgagaagaacccaatcgacttcctcgaggccaagggctacaaggaggtgaagaaggacctcatcatcaagctcccgaagtacagcctcttcgagcttgagaacggccgcaagagaatgctcgcctctgctggcgagcttcagaagggcaacgagcttgctctcccgtccaagtacgtgaacttcctctacctcgcctcccactacgagaagctcaagggctccccagaggacaacgagcaaaagcagctgttcgtcgagcagcacaagcactacctcgacgagatcatcgagcagatctccgagttctccaagcgcgtgatcctcgccgatgccaacctcgataaggtgctcagcgcctacaacaagcaccgcgataagccaattcgcgagcaggccgagaacatcatccacctcttcaccctcaccaacctcggcgctccagccgccttcaagtacttcgacaccaccatcgaccgcaagcgctacacctctaccaaggaggttctcgacgccaccctcatccaccagtctatcacaggcctctacgagacacgcatcgacctctcacaactcggcggcgatTCCGGCGGCTCCAGCGGCGGCTCATCTGGATCAGAGACACCAGGCACATCAGAGTCAGCAACACCGGAGTCCAGCGGCGGCTCATCTGGCGGCTCCAGCACACTCAATATCGAGGACGAGTACAGGCTGCATGAGACATCCAAGGAGCCTGACGTCTCCCTCGGCAGCACATGGCTCTCAGATTTCCCACAGGCCTGGGCCGAGACAGGCGGCATGGGCCTCGCCGTCCGCCAGGCGCCGCTCATCATTCCACTGAAGGCGACCTCCACACCGGTGAGCATCAAGCAGTACCCAATGTCTCAGGAGGCAAGGCTGGGCATCAAGCCACACATTCAGAGGCTCCTGGACCAGGGCATTCTGGTGCCTTGCCAGAGCCCGTGGAACACCCCTCTCCTGCCGGTGAAGAAGCCTGGCACAAATGACTACCGCCCGGTCCAGGATCTCAGGGAGGTGAACAAGCGCGTCGAGGATATCCATCCGACAGTCCCGAACCCATACAATCTCCTGTCAGGCCTCCCGCCATCTCACCAGTGGTACACCGTGCTCGACCTGAAGGATGCGTTCTTCTGCCTCAGGCTGCATCCAACAAGCCAGCCTCTCTTCGCCTTCGAGTGGCGCGATCCGGAGATGGGCATTTCAGGCCAGCTCACCTGGACACGGCTGCCACAGGGCTTCAAGAACTCTCCTACCCTCTTCAATGAGGCGCTCCATCGGGACCTGGCCGATTTCAGGATCCAGCACCCAGACCTCATTCTCCTCCAGTATGTGGACGATCTCCTGCTCGCCGCGACATCCGAGCTGGATTGCCAGCAGGGAACCCGCGCGCTGCTCCAGACACTGGGAAATCTGGGATACAGGGCATCAGCGAAGAAGGCACAGATCTGCCAGAAGCAGGTCAAGTACCTCGGCTACCTGCTCAAGGAGGGACAGAGGTGGCTGACAGAGGCAAGGAAGGAGACAGTGATGGGCCAGCCTACCCCGAAGACACCACGGCAGCTCAGGGAGTTCCTGGGCAAGGCGGGCTTCTGCCGCCTCTTCATCCCAGGATTCGCGGAGATGGCGGCGCCACTCTACCCTCTGACCAAGCCTGGCACACTGTTCAACTGGGGACCAGACCAGCAGAAGGCGTACCAGGAGATTAAGCAGGCCCTGCTCACAGCACCTGCCCTCGGCCTGCCGGACCTCACAAAGCCATTCGAGCTGTTCGTGGATGAGAAGCAGGGCTACGCGAAGGGAGTCCTGACACAGAAGCTGGGACCATGGAGGCGCCCAGTGGCCTACCTCTCCAAGAAGCTGGACCCAGTGGCTGCCGGCTGGCCTCCGTGCCTGAGGATGGTGGCGGCCATTGCCGTCCTCACCAAGGATGCCGGCAAGCTGACAATGGGCCAGCCTCTCGTCATTCTGGCGCCGCATGCGGTGGAGGCGCTCGTCAAGCAGCCACCTGATAGGTGGCTGTCCAACGCGCGCATGACCCACTACCAGGCCCTGCTCCTGGACACAGATAGGGTGCAGTTCGGCCCAGTGGTCGCCCTCAATCCTGCCACACTGCTGCCACTCCCTGAGGAGGGCCTCCAGCATAACTGCCTCGATATTCTGGCGGAGGCCCATGGAACCCGCCCTGACCTCACAGATCAGCCGCTGCCAGACGCCGATCACACCTGGTACACAGATGGCTCATCTCTCCTCCAGGAGGGCCAGAGGAAGGCCGGAGCCGCGGTGACCACAGAGACAGAGGTCATCTGGGCAAAGGCGCTCCCAGCCGGCACCTCCGCACAGAGGGCCGAGCTGATTGCACTGACACAGGCGCTCAAGATGGCCGAGGGCAAGAAGCTGAATGTGTACACCGACTCACGCTACGCCTTCGCGACAGCCCACATCCATGGAGAGATCTACAGGAGGAGGGGATGGCTCACATCTGAGGGCAAGGAGATCAAGAACAAGGATGAGATTCTCGCGCTCCTGAAGGCCCTCTTCCTGCCAAAGCGCCTGTCAATCATTCACTGCCCTGGCCATCAGAAGGGACACTCTGCGGAGGCAAGGGGAAATAGGATGGCCGACCAGGCGGCCAGGAAGGCAGCGATCACCGAGACACCGGATACCTCCACACTCCTGATTGAGAACTCCAGCCCATCAGGCGGCTCTAAGAGGACCGCCGACGGATCAGAGTTCGAGCCGAAGAAGAAGAGGAAGGTGTCCGGCGGCTCCCCGAAGAAGAAGAGGAAGGTGTCCGGCGGCTCCCCGAAGAAGAAGAGGAAAGTGTGA

Black, nSpCas9-H840A;

Blue, NLS;

Green, IGFpm1-linker-NFATC2IPp1-linker;

Purple, 33aa linker;

Orange, M-MLV RT.

>phyPE2

atggccccaaagaagaagcgcaaggtcgacaagaagtactccatcggcctcgacatcggcaccaattctgttggctgggccgtgatcaccgacgagtacaaggtgccgtccaagaagttcaaggtcctcggcaacaccgaccgccactccatcaagaagaatctcatcggcgccctgctgttcgactctggcgagacagccgaggctacaaggctcaagaggaccgctagacgcaggtacaccaggcgcaagaaccgcatctgctacctccaagagatcttctccaacgagatggccaaggtggacgacagcttcttccacaggctcgaggagagcttcctcgtcgaggaggacaagaagcacgagcgccatccgatcttcggcaacatcgtggatgaggtggcctaccacgagaagtacccgaccatctaccacctccgcaagaagctcgtcgactccaccgataaggccgacctcaggctcatctacctcgccctcgcccacatgatcaagttcaggggccacttcctcatcgagggcgacctcaacccggacaactccgatgtggacaagctgttcatccagctcgtgcagacctacaaccagctgttcgaggagaacccgatcaacgcctctggcgttgacgccaaggctattctctctgccaggctctctaagtcccgcaggctcgagaatctgatcgcccaacttccgggcgagaagaagaatggcctcttcggcaacctgatcgccctctctcttggcctcaccccgaacttcaagtccaacttcgacctcgccgaggacgccaagctccagctttccaaggacacctacgacgacgacctcgacaatctcctcgcccagattggcgatcagtacgccgatctgttcctcgccgccaagaatctctccgacgccatcctcctcagcgacatcctcagggtgaacaccgagatcaccaaggccccactctccgcctccatgatcaagaggtacgacgagcaccaccaggacctcacactcctcaaggccctcgtgagacagcagctcccagagaagtacaaggagatcttcttcgaccagtccaagaacggctacgccggctacatcgatggcggcgcttctcaagaggagttctacaagttcatcaagccgatcctcgagaagatggacggcaccgaggagctgctcgtgaagctcaatagagaggacctcctccgcaagcagcgcaccttcgataatggctccatcccgcaccagatccacctcggcgagcttcatgctatcctccgcaggcaagaggacttctacccgttcctcaaggacaaccgcgagaagattgagaagatcctcaccttccgcatcccgtactacgtgggcccgctcgccaggggcaactccaggttcgcctggatgaccagaaagtccgaggagacaatcaccccctggaacttcgaggaggtggtggataagggcgcctctgcccagtctttcatcgagcgcatgaccaacttcgacaagaacctcccgaacgagaaggtgctcccgaagcactcactcctctacgagtacttcaccgtgtacaacgagctgaccaaggtgaagtacgtgaccgaggggatgaggaagccagctttccttagcggcgagcaaaagaaggccatcgtcgacctgctgttcaagaccaaccgcaaggtgaccgtgaagcagctcaaggaggactacttcaagaaaatcgagtgcttcgactccgtcgagatctccggcgtcgaggataggttcaatgcctccctcgggacctaccacgacctcctcaagattatcaaggacaaggacttcctcgacaacgaggagaacgaggacatcctcgaggacatcgtgctcaccctcaccctcttcgaggaccgcgagatgatcgaggagcgcctcaagacatacgcccacctcttcgacgacaaggtgatgaagcagctgaagcgcaggcgctataccggctggggcaggctctctaggaagctcatcaacggcatccgcgacaagcagtccggcaagacgatcctcgacttcctcaagtccgacggcttcgccaaccgcaacttcatgcagctcatccacgacgactccctcaccttcaaggaggacatccaaaaggcccaggtgtccggccaaggcgattccctccatgaacatatcgccaatctcgccggctccccggctatcaagaagggcattctccagaccgtgaaggtggtggacgagctggtgaaggtgatgggcaggcacaagccagagaacatcgtgatcgagatggcccgcgagaaccagaccacacagaagggccaaaagaactcccgcgagcgcatgaagaggatcgaggagggcattaaggagctgggctcccagatcctcaaggagcacccagtcgagaacacccagctccagaacgagaagctctacctctactacctccagaacggccgcgacatgtacgtggaccaagagctggacatcaaccgcctctccgactacgacgtggacGCtattgtgccgcagtccttcctgaaggacgactccatcgacaacaaggtgctcacccgctccgacaagaacaggggcaagtccgataacgtgccgtccgaagaggtcgtcaagaagatgaagaactactggcgccagctcctcaacgccaagctcatcacccagaggaagttcgacaacctcaccaaggccgagagaggcggcctttccgagcttgataaggccggcttcatcaagcgccagctcgtcgagacacgccagatcacaaagcacgtggcccagatcctcgactcccgcatgaacaccaagtacgacgagaacgacaagctcatccgcgaggtgaaggtcatcaccctcaagtccaagctcgtgtccgacttccgcaaggacttccagttctacaaggtgcgcgagatcaacaactaccaccacgcccacgacgcctacctcaatgccgtggtgggcacagccctcatcaagaagtacccaaagctcgagtccgagttcgtgtacggcgactacaaggtgtacgacgtgcgcaagatgatcgccaagtccgagcaagagatcggcaaggcgaccgccaagtacttcttctactccaacatcatgaatttcttcaagaccgagatcacgctcgccaacggcgagattaggaagaggccgctcatcgagacaaacggcgagacaggcgagatcgtgtgggacaagggcagggatttcgccacagtgcgcaaggtgctctccatgccgcaagtgaacatcgtgaagaagaccgaggttcagaccggcggcttctccaaggagtccatcctcccaaagcgcaactccgacaagctgatcgcccgcaagaaggactgggacccgaagaagtatggcggcttcgattctccgaccgtggcctactctgtgctcgtggttgccaaggtcgagaagggcaagagcaagaagctcaagtccgtcaaggagctgctgggcatcacgatcatggagcgcagcagcttcgagaagaacccaatcgacttcctcgaggccaagggctacaaggaggtgaagaaggacctcatcatcaagctcccgaagtacagcctcttcgagcttgagaacggccgcaagagaatgctcgcctctgctggcgagcttcagaagggcaacgagcttgctctcccgtccaagtacgtgaacttcctctacctcgcctcccactacgagaagctcaagggctccccagaggacaacgagcaaaagcagctgttcgtcgagcagcacaagcactacctcgacgagatcatcgagcagatctccgagttctccaagcgcgtgatcctcgccgatgccaacctcgataaggtgctcagcgcctacaacaagcaccgcgataagccaattcgcgagcaggccgagaacatcatccacctcttcaccctcaccaacctcggcgctccagccgccttcaagtacttcgacaccaccatcgaccgcaagcgctacacctctaccaaggaggttctcgacgccaccctcatccaccagtctatcacaggcctctacgagacacgcatcgacctctcacaactcggcggcgatTCAGGCGGGAGTTCAGGAGGTAGTAGCGGCAGCGAAACCCCAGGGACATCAGAATCAGCGACACCAGAAAGCAGTGGAGGAAGTAGCGGCGGATCTATGGCGATGCAGATGCAACTGGAGGCTAACGCAGACACCTCGGTTGAGGAAGAGAGTTTCGGACCGCAGCCAATCTCCAGGCTGGAGCAATGCGGCATCAACGCGAATGATGTGAAGAAGCTGGAGGAGGCTGGCTTTCACACAGTGGAAGCCGTCGCGTACGCTCCGAAGAAGGAGCTGATCAACATCAAGGGAATCAGCGAAGCAAAGGCCGACAAAATTCTTGCGGAGGCGGCCAAGTTGGTCCCGATGGGCTTCACCACAGCCACCGAATTTCATCAGAGGCGCTCCGAGATCATTCAAATCACTACGGGCAGCAAGGAACTGGATAAGCTCCTCCAGTCAGGAGGTTCCAGCGGCGGGTCATCTGGGTCCGAGACACCAGGCACGAGCGAGTCAGCAACCCCAGAAAGTAGCGGTGGTAGTAGTGGTGGCAGTACACTCAATATCGAGGACGAGTACAGGCTGCATGAGACATCCAAGGAGCCTGACGTCTCCCTCGGCAGCACATGGCTCTCAGATTTCCCACAGGCCTGGGCCGAGACAGGCGGCATGGGCCTCGCCGTCCGCCAGGCGCCGCTCATCATTCCACTGAAGGCGACCTCCACACCGGTGAGCATCAAGCAGTACCCAATGTCTCAGGAGGCAAGGCTGGGCATCAAGCCACACATTCAGAGGCTCCTGGACCAGGGCATTCTGGTGCCTTGCCAGAGCCCGTGGAACACCCCTCTCCTGCCGGTGAAGAAGCCTGGCACAAATGACTACCGCCCGGTCCAGGATCTCAGGGAGGTGAACAAGCGCGTCGAGGATATCCATCCGACAGTCCCGAACCCATACAATCTCCTGTCAGGCCTCCCGCCATCTCACCAGTGGTACACCGTGCTCGACCTGAAGGATGCGTTCTTCTGCCTCAGGCTGCATCCAACAAGCCAGCCTCTCTTCGCCTTCGAGTGGCGCGATCCGGAGATGGGCATTTCAGGCCAGCTCACCTGGACACGGCTGCCACAGGGCTTCAAGAACTCTCCTACCCTCTTCAATGAGGCGCTCCATCGGGACCTGGCCGATTTCAGGATCCAGCACCCAGACCTCATTCTCCTCCAGTATGTGGACGATCTCCTGCTCGCCGCGACATCCGAGCTGGATTGCCAGCAGGGAACCCGCGCGCTGCTCCAGACACTGGGAAATCTGGGATACAGGGCATCAGCGAAGAAGGCACAGATCTGCCAGAAGCAGGTCAAGTACCTCGGCTACCTGCTCAAGGAGGGACAGAGGTGGCTGACAGAGGCAAGGAAGGAGACAGTGATGGGCCAGCCTACCCCGAAGACACCACGGCAGCTCAGGGAGTTCCTGGGCAAGGCGGGCTTCTGCCGCCTCTTCATCCCAGGATTCGCGGAGATGGCGGCGCCACTCTACCCTCTGACCAAGCCTGGCACACTGTTCAACTGGGGACCAGACCAGCAGAAGGCGTACCAGGAGATTAAGCAGGCCCTGCTCACAGCACCTGCCCTCGGCCTGCCGGACCTCACAAAGCCATTCGAGCTGTTCGTGGATGAGAAGCAGGGCTACGCGAAGGGAGTCCTGACACAGAAGCTGGGACCATGGAGGCGCCCAGTGGCCTACCTCTCCAAGAAGCTGGACCCAGTGGCTGCCGGCTGGCCTCCGTGCCTGAGGATGGTGGCGGCCATTGCCGTCCTCACCAAGGATGCCGGCAAGCTGACAATGGGCCAGCCTCTCGTCATTCTGGCGCCGCATGCGGTGGAGGCGCTCGTCAAGCAGCCACCTGATAGGTGGCTGTCCAACGCGCGCATGACCCACTACCAGGCCCTGCTCCTGGACACAGATAGGGTGCAGTTCGGCCCAGTGGTCGCCCTCAATCCTGCCACACTGCTGCCACTCCCTGAGGAGGGCCTCCAGCATAACTGCCTCGATATTCTGGCGGAGGCCCATGGAACCCGCCCTGACCTCACAGATCAGCCGCTGCCAGACGCCGATCACACCTGGTACACAGATGGCTCATCTCTCCTCCAGGAGGGCCAGAGGAAGGCCGGAGCCGCGGTGACCACAGAGACAGAGGTCATCTGGGCAAAGGCGCTCCCAGCCGGCACCTCCGCACAGAGGGCCGAGCTGATTGCACTGACACAGGCGCTCAAGATGGCCGAGGGCAAGAAGCTGAATGTGTACACCGACTCACGCTACGCCTTCGCGACAGCCCACATCCATGGAGAGATCTACAGGAGGAGGGGATGGCTCACATCTGAGGGCAAGGAGATCAAGAACAAGGATGAGATTCTCGCGCTCCTGAAGGCCCTCTTCCTGCCAAAGCGCCTGTCAATCATTCACTGCCCTGGCCATCAGAAGGGACACTCTGCGGAGGCAAGGGGAAATAGGATGGCCGACCAGGCGGCCAGGAAGGCAGCGATCACCGAGACACCGGATACCTCCACACTCCTGATTGAGAACTCCAGCCCATCAGGCGGCTCTAAGAGGACCGCCGACGGATCAGAGTTCGAGCCGAAGAAGAAGAGGAAGGTGTCCGGCGGCTCCCCGAAGAAGAAGAGGAAGGTGTCCGGCGGCTCCCCGAAGAAGAAGAGGAAAGTGTGA

Black, nSpCas9-H840A;

Blue, NLS;

Green, linker-ssDBD-linker;

Orange, M-MLV RT.

>epegRNA cassette in enpPE2

ATGGAGTCAAAGATTCAAATAGAGGACCTAACAGAACTCGCCGTAAAGACTGGCGAACAGTTCATACAGAGTCTCTTACGACTCAATGACAAGAAGAAAATCTTCGTCAACATGGTGGAGCACGACACACTTGTCTACTCCAAAAATATCAAAGATACAGTCTCAGAAGACCAAAGGGCAATTGAGACTTTTCAACAAAGGGTAATATCCGGAAACCTCCTCGGATTCCATTGCCCAGCTATCTGTCACTTTATTGTGAAGATAGTGGAAAAGGAAGGTGGCTCCTACAAATGCCATCATTGCGATAAAGGAAAGGCCATCGTTGAAGATGCCTCTGCCGACAGTGGTCCCAAAGATGGACCCCCACCCACGAGGAGCATCGTGGAAAAAGAAGACGTTCCAACCACGTCTTCAAAGCAAGTGGATTGATGTGATTGGCAGACATACTGTCCCACAAATGAAGATGGAATCTGTAAAAGAAAACGCGTGAAATAATGCGTCTGACAAAGGTTAGGTCGGCTGCCTTTAATCAATACCAAAGTGGTCCCTACCACGATGGAAAAACTGTGCAGTCGGTTTGGCTTTTTCTGACGAACAAATAAGATTCGTGGCCGACAGGTGGGGGTCCACCATGTGAAGGCATCTTCAGACTCCAATAATGGAGCAATGACGTAAGGGCTTACGAAATAAGTAAGGGTAGTTTGGGAAATGTCCACTCACCCGTCAGTCTATAAATACTTAGCCCCTCCCTCATTGTTAAGGGAGCAAAATCTCAGAGAGATAGTCCTAGAGAGAGAAAGAGAGCAAGTAGCCTAGAAGTAGTCAAGGCGGCGAAGTATTCAGGCACGTGGCCAGGAAGAAGAAAAGCCAAGACGACGAAAACAGGTAAGAGCTAAGCATCTAGAAAGTTGAAAACAATCTTCAAAAGTCCCACATCGCTTAGATAAGAAAACGAAGCTGAGTTTATATACAGCTAGAGTCGAAGTAGTGATTGAACAAAGCACCAGTGGTCTAGTGGTAGAATAGTACCCTGCCACGGTACAGACCCGGGTTCGATTCCCGGCTGGTGCA……GGCCGGCATGGTCCCAGCCTCCTCGCTGGCGCCGGCTGGGCAACATGCTTCGGCATGGCGAATGGGACTTTTTTTTGATATCTCCGGGGCTAATTGAATATGAAGATGAAGATGAAATATTTGGTGTGTCAAATAAAAAGCTGGTGTGCTTAAGTTTGTGTTTTTTTCTTGGCTTGTTGTGTTATGAATTTGTGGCTTTTTCTAATATTAAATGAATGTAAGATCTCATTATAATGAATAAACAAATGTTTCTATAATCCATTGTGAATGTTTTGTTGGATCTCTTCTGCAGCATATAACTACTGTATGTGCTATGGTATGGACTATGGAATATGATTAAAGATAAG

Yellow, CaMV 35S enhancer;

Orange, CmYLCV promoter;

Red, shortened U6-26 promoter;

Green, tRNA (Gly) sequence;

……, epegRNA sequence;

Black, HDV;

Purple, poly T terminator;

Blue, AtHSP18.2 terminator.
