# Supplementary material for: Development of a highly efficient prime editor 2 system in plants
Source: Genome Biol. 2022 Jul 25;23:161. doi: 10.1186/s13059-022-02730-x (PMC9310484; doi:10.1186/s13059-022-02730-x)
Supplement: Supplementary file 2 — Additional file 2. Review history. [file 13059_2022_2730_MOESM2_ESM.docx]

Review History

**First round of review**

**Reviewer 1**

**Are you able to assess all statistics in the manuscript, including the appropriateness of statistical tests used?** There are no statistics in the manuscript.

**Comments to author:**

Low editing efficiency has restrained the original PE systems from being used broadly in plants. In this paper, authors present the results quite inspiring and exciting and the tools described in this paper will encourage researchers to use PEs for precise genome editing.

Minor revisions
1. In the title, 'optimized' should be used instead of 'optimizing'. The reason: if prime editor 2 systems are already highly efficient, authors have no necessity to optimize them.
2. 'epegRNA-evopreQ1' should be revised to 'pegRNA-evopreQ1'
3. Names of proteins and prime editing systems should be carefully discerned, e.g., PE4max should be used to refer to the prime editing system rather than protein architecture. I suggest using PEmax-MLH1dn instead of PE4max when referring to protein architecture. PEmax-MLH1dn and pegRNAs constitute PE4max system, and PEmax-MLH1dn, pegRNAs, and nicking sgRNAs constitute PE5max system.
4. Please check the sequence annotations of U6 and tRNA(Gly)

**Reviewer 2**

**Are you able to assess all statistics in the manuscript, including the appropriateness of statistical tests used?** Yes, and I have assessed the statistics in my report.

**Comments to author:**

As an important gene editing tool, prime editing (PE) system is difficult to apply in plant breeding due to its low editing efficiency. In this manuscript, Li et al. reported the combination of the CaMV35S-CmYLCV-U6::epegRNA-evopreQ1 and pPE2max can greatly improved the PE efficiencies in stably transformed callus cells and transgenic plants. A wide variety mutation types were successfully introduced in rice by using the optimized system. This system provides a significant help for the better application of PE system to agricultural practice. I think the manuscript is up to Genome Biology standard and recommended for publication.

I only have some minor comments:
1. As author pointed out that CaMV35S-CmYLCV-U6::epegRNA-evopreQ1 was essential to improve the PE efficiency in rice, is there any speciality of this component. Further discussions should be needed.
2. It is recommended to use a uniform alignment format for the full text.
3. It is better to cite the recently reported article about the structured RNA motifs' application from Caixia Gao's work.

**Reviewer 3**

**Are you able to assess all statistics in the manuscript, including the appropriateness of statistical tests used?** Yes, and I have assessed the statistics in my report.

**Comments to author:**

Low efficiency limited the potential application of prime editing systems in plant science and crop improvement. To improve editing ability, Li et al. firstly validated the previous documented strategies for optimization of the plant prime editor 2 (pPE2) system and tested their editing efficiencies in rice callus and stably transformed plants. Furthermore, an enhanced pPE2 (enpPE2) system was developed by stacking the optimized strategies. Compared to the pPE2 system, enpPE2 significantly increased the prime editing efficiency, providing a robust and powerful prime editing tool for precise modification of the plant genome. Overall, it is very helpful to the plant genome editing field to evaluate the previous optimized strategies in plant prime editing. This work represents a significant advance over previously published studies. And the enhanced prime editing system will certainly benefit the application of prime editing in crop improvement. The methods were well described and appropriate to the aims of the study. However, I have the following major and minor concerns need to be addressed before acceptance of this manuscript for publication in Genome Biology.
Major concerns
1. In Abstract section, 'An enhanced pPE2 (enpPE2) system was constructed by stacking feasible optimization strategies', it would be better to revise into 'An enhanced pPE2 (enpPE2) system was developed by stacking various optimization strategies such as …'. Furthermore, it would be good to describe the strategies in order to be more informative for the readers. Moreover, 'enpPE2 increased the prime editing efficiency by an average of 413.57-fold and a maximum of 1332.23-fold in rice cells', I didn't see any results to support this claim. Here, I would like to suggest the authors to present the editing outcome (or increased efficiency) based on the obtained independent stable lines rather than the sequence results of the rice calli.
2. It seems that the T0 plants in Table 1 (Page 16) are not independent lines, this will lead to overestimation of the precise editing outcome and will be misleading to the readers, because the number of plants were much more than transformed calli. I strongly suggest that the overall efficiency for each experiment or biological repeat should be calculated based on the obtained independent events.
Minor concerns
1. Title 'Optimizing highly efficient prime editor 2 systems in plants', it would be better to change into 'Development of a highly efficient prime editor 2 system in plants'
2. Page 5, line 56, 'silent' should be 'synonymous'.
3. Please cite the recent publication by Li et al (2022) in Molecular Plant.
Li et al. 2022. Multiplex precision gene editing by a surrogate prime editor in rice. Molecular Plant, DOI: <https://doi.org/10.1016/j.molp.2022.05.009>

**Authors Response**

**Point-by-point responses to the reviewers’ comments:**

Editor comments:
Q1. As you will see, the reports are broadly favorable, so we are interested in publishing the manuscript, but we feel that the issues raised must be addressed in full, in the form of a revised manuscript, before we make a firm commitment to publication. When revising your manuscript, please ensure that all the points raised by the referees are addressed.
***Response: We sincerely thank the editor and the referees for the kind encouragement and valuable suggestions. We agree with all comments provided by the referees, and the manuscript was thoroughly revised accordingly. All concerns raised by the reviewers were carefully and fully responded to. We hope that the manuscript has been improved with the help from your and the referees.***

Q2. When revising the manuscript, please also ensure that the manuscript is formatted according to our instructions and that editable figures are provided with the revised manuscript. Please see https://genomebiology.biomedcentral.com/submission-guidelines/preparing-your-manuscript/short-report for further details. We also require all data to be deposited to a relevant repository prior to resubmission, although you may supply a reviewer access token if desired. Please note that if we decide to publish your manuscript we will require that all data be made publicly accessible and the 'live' accession numbers included in a separate Availability of Data and Materials section of the manuscript.
***Response: We appreciate the comment. The manuscript and the figure have been formatted according to the instructions. Following the suggestion, we have double checked the active status of the deposited data under the accession numbers.***
 
Reviewer #1:
Minor revisions
Q1. In the title, 'optimized' should be used instead of 'optimizing'. The reason: if prime editor 2 systems are already highly efficient, authors have no necessity to optimize them.
***Response: We apologize for the misleading title. The title has been revised with the help of the reviewers.***

Q2. 'epegRNA-evopreQ1' should be revised to 'pegRNA-evopreQ1'
***Response: Thank you for the suggestion. All epegRNA-evopreQ1 has been revised accordingly.***

Q3. Names of proteins and prime editing systems should be carefully discerned, e.g., PE4max should be used to refer to the prime editing system rather than protein architecture. I suggest using PEmax-MLH1dn instead of PE4max when referring to protein architecture. PEmax-MLH1dn and pegRNAs constitute PE4max system, and PEmax-MLH1dn, pegRNAs, and nicking sgRNAs constitute PE5max system.
***Response: We greatly appreciate the comment. The names of prime editor proteins and prime editing systems were somehow confused in the manuscript. According to the suggestion, we carefully replaced the PE2max and PE4max proteins to PEmax and PEmax-MLHdn, respectively, in the main text, figures, tables and supplementary materials. We hope that the description of the prime editors and prime editing system is more precise in the revised version.***

Q4. Please check the sequence annotations of U6 and tRNA(Gly)
***Response: Thank you for noting the mistakes. The sequence annotations have been corrected in the manuscript.***

Reviewer #2:
Q1. As author pointed out that CaMV35S-CmYLCV-U6::epegRNA-evopreQ1 was essential to improve the PE efficiency in rice, is there any speciality of this component. Further discussions should be needed.
***Response: Thank you for your kind comment. The CaMV35S-CmYLCV-U6 promoter is a chimeric promoter combining the elements of polymerase II and III promoters. It may have binary characteristics of the two types of promoters and is thus more effective for expressing noncanonical small RNAs with relatively long sequences, such as pegRNAs. Two reports have revealed that prime editing could be enhanced by replacing pol III promoters with the U6 composite promoter in maize. Our results showed that this strategy could further improve the editing activity of PEs optimized via other functional strategies in rice. As requested, the related discussion was added on Page 7 line 9 to 13 of the revised manuscript.***

Q2. It is recommended to use a uniform alignment format for the full text.
***Response: We apologize for the flaws in the text. In this version, we carefully revised the manuscript. The full text was formatted with justification alignment. To improve the writing quality, the professional English service “American Journal Experts” was employed according to suggestion of the Journal guideline to correct any mistakes in the language and format of the text.***

Q3. It is better to cite the recently reported article about the structured RNA motifs' application from Caixia Gao's work.
***Response: Thank you for the comment. According to the suggestion, the appropriate literature was discussed and cited in Page 9 line 12 to 15 of the revised manuscript.***
 
Reviewer #3:
Major concerns
Q1. In Abstract section, 'An enhanced pPE2 (enpPE2) system was constructed by stacking feasible optimization strategies', it would be better to revise into 'An enhanced pPE2 (enpPE2) system was developed by stacking various optimization strategies such as …'. Furthermore, it would be good to describe the strategies in order to be more informative for the readers. Moreover, 'enpPE2 increased the prime editing efficiency by an average of 413.57-fold and a maximum of 1332.23-fold in rice cells', I didn't see any results to support this claim. Here, I would like to suggest the authors to present the editing outcome (or increased efficiency) based on the obtained independent stable lines rather than the sequence results of the rice calli.
***Response: We sincerely appreciate the comment. The sentence starting with “An enhanced pPE2” was revised accordingly, and the detailed strategies, including “updating the PE architecture to PEmax and expressing engineered pegRNA with a structured motif under the control of a composite promoter”, were described following the suggestion. Regarding the fold increases in editing efficiency, we apologize for the mistakes in the previous version of the manuscript. We should average the editing frequencies at different sites to calculate the fold of activity enhancement instead of simply averaging the fold changes at the targets. Recalculation showed that enpPE2 presented an average editing frequency of 25.65%, corresponding to an ~43.47-fold efficiency increase (maximum ~1280-fold increase from 0.02% to 25.60% at the ACC-T site) relative to the average 0.59% frequency of the unmodified pPE2 system. These data have been corrected in Page 7 line 16 to 19 of the main text. According to the comment, we present the editing efficiency of independent stable lines of both unmodified pPE2 and optimized enpPE2 systems to support the activity enhancement. We hope that the revised Abstract is more informative to readers under the 100 words limitation required by Genome biology.***

Q2. It seems that the T0 plants in Table 1 (Page 16) are not independent lines, this will lead to overestimation of the precise editing outcome and will be misleading to the readers, because the number of plants were much more than transformed calli. I strongly suggest that the overall efficiency for each experiment or biological repeat should be calculated based on the obtained independent events.
***Response: We apologize for our unclear description. Actually, all T0 plants analyzed in the manuscript are independent lines. All data from stable transgenic plants were generated and calculated based on independent transformation events (lines). The idea that “the number of plants were much more than transformed calli” might originate from the potential misleading statement that “In each resistance event, three to four calli were selected for plant regeneration under 25 mg/L hygromycin selection” in the Methods section. In fact, “three to four” did not refer to the transformed calli per vector but to the biological repeats of material from a single selection event to increase the frequency of regeneration. In our experiment, more than 500 healthy secondary calli were selected and infected with Agrobacterium per vector. After infection, the calli were dried and allowed to recover for 5 days. For each vector, approximately 300~350 recovered calli were selected under 50 mg/L hygromycin treatment. After 4 weeks of selection, a group of resistant calli grown from a single original callus was considered as one independent transformation (selection) event. Normally, >250 independent selection events could be obtained per vector transformation at this step. Among them, 200 well-growth independent selection events per vector were chosen for further regeneration. Since there were many calli in a single independent selection event, only three to four intermediate-sized, solid, and yellowish calli were chosen from them and transformed together as one independent event for plant regeneration under 25 mg/L hygromycin selection. Although several plants might have been regenerated in one regeneration event, only a single plant was rooted as an independent line. For each vector, we generally obtained more than 50 independent transgenic lines. Among them, 48 independent lines were randomly selected for further analysis. To avoid misunderstanding, we rewrote the corresponding part of the Methods section. In addition, we want to declare that the assessments of efficiency for each experiment in the manuscript, not only in regenerated plants but also in resistant calli, were performed and analyzed based on independent events or biological independent repeats.***

Minor concerns
Q1. Title 'Optimizing highly efficient prime editor 2 systems in plants', it would be better to change into 'Development of a highly efficient prime editor 2 system in plants'
***Response: Thank you for the comment. The title has been revised with the help of the reviewers.***

Q2. Page 5, line 56, 'silent' should be 'synonymous'.
***Response: Thank you for the comment. The word has been corrected accordingly.***

Q3. Please cite the recent publication by Li et al (2022) in Molecular Plant.
Li et al. 2022. Multiplex precision gene editing by a surrogate prime editor in rice. Molecular Plant, DOI: https://doi.org/10.1016/j.molp.2022.05.009
***Response: We appreciate the suggestion. The literature was discussed and cited in the revised manuscript.***

**Second round of review**

**Reviewer 3**

Manuscript Number: GBIO-D-22-00398R1
Title: Development of a highly efficient prime editor 2 system in plants
In the revised manuscript, the authors have addressed most of my major concerns. However, one issue needs to be addressed before acceptance for publication of this manuscript in Genome Biology.
1. In the revised manuscript, line 20-22, Page 7, '…64.58% to 77.08% of the T0 line plants carried targeted mutations introduced by enpPE2…(Table 1)' . Here, I am wondering if these lines carrying the targeted mutations are precise modifications or mutations including both precise modifications and desired mutations. Sometimes, during prime editing, you may get the lines carrying the desired mutations, but not a precise modification exactly as you designed. The authors should further clarify the description of the above result in order to give the readers a clear view.
2. Page 16, Line 36-40, in the reference list, for the newly added references, please add DOI number at the end of each reference.

**Authors Response**

**Point-by-point responses to the reviewers’ comments:**

Comment 1. In the revised manuscript, the authors have addressed most of my major concerns. However, one issue needs to be addressed before acceptance for publication of this manuscript in Genome Biology.
In the revised manuscript, line 20-22, Page 7, '…64.58% to 77.08% of the T0 line plants carried targeted mutations introduced by enpPE2…(Table 1)' . Here, I am wondering if these lines carrying the targeted mutations are precise modifications or mutations including both precise modifications and desired mutations. Sometimes, during prime editing, you may get the lines carrying the desired mutations, but not a precise modification exactly as you designed. The authors should further clarify the description of the above result in order to give the readers a clear view.
***Response 1: We sincerely appreciate for the comments and suggestions from the Reviewer #3 during the reviewing process. We agree with the reviewer that prime editing may generate modified plants simultaneously consisting the desired mutations and byproduct mutations. Fortunately, the editing on the four sites described in the sentence exhibited limited frequency of byproduct mutations. The presented edited plants, which account 64.58% to 77.08% of the T0 line plants, only carried precisely desired mutations but no byproducts. The plants harboring unprecise modification were described in the last sentence of the paragraph and present at different column of Table 1. According to the suggestion, we have revised the two sentences to avoid potential confusions.***

Comment 2. Page 16, Line 36-40, in the reference list, for the newly added references, please add DOI number at the end of each reference.
***Response 2：Thank you for the comment. The DOI numbers have been added to the reference.***
